# Associations between comorbidities, their treatment and survival in patients with interstitial lung diseases – a claims data analysis

**DOI:** 10.1186/s12931-018-0769-0

**Published:** 2018-04-25

**Authors:** Larissa Schwarzkopf, Sabine Witt, Julia Waelscher, Markus Polke, Michael Kreuter

**Affiliations:** 1 Helmholtz Zentrum München – German Research Center for Environmental Health (GmbH), Institute of Health Economics and Health Care Management, Comprehensive Pneumology Center Munich (CPC-M), Member of the German Center for Lung Research (DZL), Ingolstaedter Landstrasse 1, 85764 Neuherberg, Germany; 2 Center for Interstitial and Rare Lung Diseases, Pneumology and Respiratory Critical Care Medicine, Thoraxklinik, University of Heidelberg, Translational Lung Research Center Heidelberg (TLRC), Member of the German Center for Lung Research (DZL), Roentgenstrasse 1, 69126 Heidelberg, Germany

**Keywords:** Administrative data, Pharmaceutical management, Drugs, Mortality, Diffuse parenchymal lung disease, Multi-morbidity, Germany, Statutory Health Insurance

## Abstract

**Background:**

Interstitial lung diseases (ILDs) are associated with a high burden of disease. However, data on the prognostic impact of comorbidities and comorbidity-related pharmaceutical treatments in patients with various ILDs remain sparse.

**Methods:**

Using longitudinal claims data from a German Statutory Health Insurance Fund, we assessed comorbidity in ILD subtypes and associated drug treatments. Baseline comorbidity was assessed via the Elixhauser Comorbidity Index that was amended by ILD-relevant conditions. Drug treatment was assessed on the substance level using the ATC-codes of drugs prescribed at the time of ILD diagnosis. Subsequently, the comorbid conditions (main analysis) and pharmaceutical substances (secondary analysis) with a meaningful association to survival were identified for the complete ILD cohort and within the subtype strata. For this, we applied multivariate Cox models using a LASSO selection process and visualized the findings within comorbidomes.

**Results:**

In the 36,821 patients with ILDs, chronic obstructive pulmonary disease (COPD), arterial hypertension, and ischaemic heart disease (IHD) were the most prevalent comorbidities. The majority of patients with cardiovascular diseases received pharmaceutical treatment, while, in other relevant comorbidities, treatment quotas were low (COPD 46%, gastro-oesophageal reflux disease 65%). Comorbidities had a clinically meaningful detrimental effect on survival that tended to be more pronounced in the case of untreated conditions (e.g. hazard ratios for treated IHD 0.97 vs. 1.33 for untreated IHD). Moreover, comorbidity impact varied substantially between distinct subtypes.

**Conclusions:**

Our analyses suggest that comorbid conditions and their treatment profile significantly affect mortality in various ILDs. Therefore, comprehensive comorbidity assessment and management remains important in any ILD.

**Electronic supplementary material:**

The online version of this article (10.1186/s12931-018-0769-0) contains supplementary material, which is available to authorized users.

## Background

Interstitial lung diseases (ILDs) comprise around 200 different rare diseases that are mostly associated with high mortality but are heterogeneous regarding aetiology, prognosis, and treatment [1].

Recent clinical trials have created a sound basis for improved pharmaceutical treatment of patients with idiopathic pulmonary fibrosis (IPF) [2, 3]. However, the transferability of corresponding findings to routine care settings remains slightly uncertain [4]. This has also been highlighted in other diseases such as chronic obstructive pulmonary disease (COPD), in which comorbidities play a significant role in management decisions and clinical outcome [5]. Assuming that a similar interaction between comorbidity and outcomes in various ILD subtypes exists, more research is currently being conducted. These investigations are urgently recommended because the relationships between an index disease (here: ILD) and comorbidities include conditions that occur incidentally (e.g. arterial hypertension) as well as potential risk factors (e.g. gastro-oesophageal reflux) or complications respectively sequelae of the index disease (e.g. pulmonary hypertension). These different interaction modes therefore introduce a high level of complexity [6].

Available evidence suggests that comorbidities substantially affect disease progression and survival in IPF [7, 8], but information on other ILD subtypes is sparse [9, 10]. Moreover, previous research has often focused on distinct conditions [11–14] instead of the patients’ entire comorbidity burden [15–17] and, consequently, has tended to disregard the effects of pharmaceutical comorbidity management [8, 10, 18–21]. Against this background, we assessed the baseline comorbidity profile of patients diagnosed with different ILD subtypes with a particular emphasis on the impact of distinct comorbid conditions and their respective pharmaceutical treatments on mortality. Since this study is the first to elucidate the associations of treated vs. untreated comorbidity and mortality in distinct ILD subtypes this new aspect may improve patient-centred ILD management.

## Methods

### Data set and sample selection

We performed a retrospective analysis of anonymized 2009–2014 patient-level insurance claims data provided by the Scientific Institute of AOK Statutory Health Insurance funds (WIdO). AOK insures about 30% of the German resident population, and the patient clientele is considered to be representative for Germany [22].

Data analyses were conducted according to national data protection laws, and AOK Bavaria approved the use of their data for the following analyses. As we performed a retrospective analysis of completely anonymised data, the consultation of an ethics committee was not required for this kind of analysis [23]. Data included in- and outpatient diagnoses based on ICD-10 codes, procedures undertaken based on the International Classification of Procedures in Medicine for inpatients, item codes from the schedule of fees for the outpatient sector, as well as information on age and gender.

Our initial dataset contained all adult insurees with a diagnosis of (a) idiopathic interstitial pneumonia (IIP) [J84.1], (b) other fibrosing ILDs (OFI) [J84.0, J84.8, J84.9, D48.1], (c) sarcoidosis (SARC) [D86.0–D86.9], (d) drug-associated ILDs (DAI) [J70.2–J70.4], (e) pneumoconiosis (PNE) [J62.0–J62.8, J63.0–J63.8], (f) radiation-associated pneumonitis (RAP) [J70.1], (g) eosinophilic pneumonia (EPP) [J82], (h) hypersensitivity pneumonitis (HP) [J67.9] and (i) connective tissue-associated ILD (CTD) [J99.1].

As a reference for our selection algorithm, we transferred the ICD-9-based validation algorithm of Esposito et al. -  that yielded a positive predictive value of 83.3% in IPF-patients [24] - into ICD-10-codes. To avoid false-positive diagnoses by non-specialists, we excluded individuals without confirmed outpatient diagnoses from a pulmonologist, an internal specialist, or a rheumatologist (for the latter CTD only) and without any inpatient diagnosis. Subsequently, other individuals who did not undergo at least one relevant diagnostic procedure (bronchoscopy, computerized tomography of the lungs (CT), pulmonary function testing, and assessment of autoantibodies) during the visit of the diagnosis were also excluded. Likewise, individuals with implausible diagnostic patterns (exclusion of ILD after confirmed diagnosis, radiation-associated pneumonitis without previous/concurrent diagnosis of malignancy, CTD without previous/concurrent diagnosis of autoimmune disease) were excluded.

**Fig. 1:**
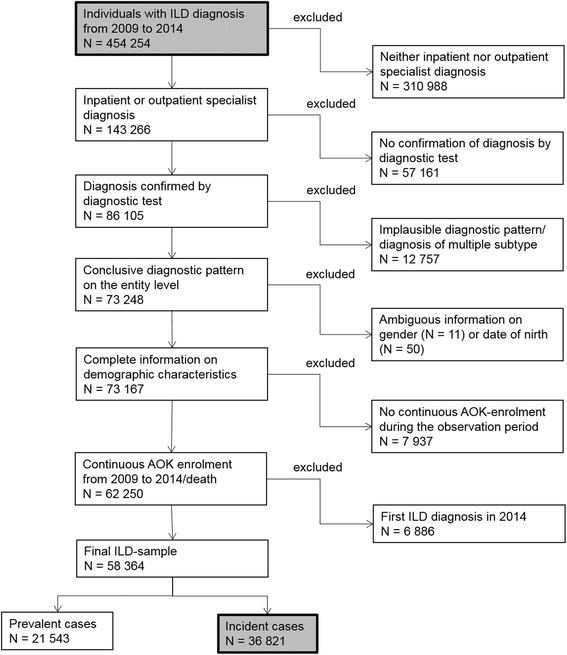
Consort diagram of sample selection

According to Walsh et al., the agreement on distinct ILD subtypes between different specialists and specialised ILD teams is limited [25]. Accordingly discrepant diagnoses of a patient’s ILD impedes a precise assessment of subtype specific comorbidity profiles. Therefore, in the next step, we excluded individuals assigned to different ILDs simultaneously. In addition, we omitted patients with inconclusive information on gender or date of birth and individuals with an interrupted sequence of AOK enrolment. Finally, patients with a first ILD diagnosis in 2014 were excluded, leaving 58,364 individuals. Those with a minimum observation period of 12 months before ILD diagnosis were classified as incident and the remainder as prevalent cases (Fig. 1).

### Assessment of variables with time reference

Portrayed as the difference between the date of diagnosis and the date of death or the end of the observation period, survival was our primary outcome parameter. Owing to data protection laws, available information contained only the month and year of death. Thus, the date of death was set on the 15th of each month for individuals deceased before 31 December 2014. Individuals surviving beyond this date were considered as censored.

Within the German health care system, outpatient diagnoses are not reported with their exact date but per quarter of the year. To define the date of diagnosis, we first assessed the quarter within which the first ILD diagnosis was performed (QDiag) and, subsequently, chose the middle of each quarter. Given that the thus defined date of diagnosis is to some extent imprecise for some patients, the date of diagnosis might be before the date of death. To avoid the exclusion of those patients who have a survival time of up to 0 days, we assigned them a fictional survival of 1 week.

### Assessment of comorbidity and medication profiles

The comorbidity burden at baseline was operationalized via the Elixhauser Index (EI) [26]. The EI was implemented using the ICD-10 coding algorithm of Quan et al. [27]. EI distinguishes between hypertension and diabetes with and without complications. Individuals with codes for both forms were assigned to the condition with complications. Additionally, the EI was slightly modified to reflect the ILD-related comorbidity more precisely. First, ‘pulmonary hypertension’ (PH) was excluded from the EI domain ‘pulmonary circulation disorders’ and analysed as a separate condition; secondly ‘lung cancer’ was removed from the EI domain ‘solid tumour without metastases’ and analysed as a separate condition as well. We also considered the non-EI conditions gastro-oesophageal reflux disease (GERD), obstructive sleep apnoea syndrome (OSAS), ischaemic heart disease (IHD), and thromboembolism based on previous evidence on their ILD relevance [15, 16, 28]. Each patient with at least one corresponding inpatient or one confirmed outpatient diagnostic code during QDiag was classified as suffering from that condition.

Comorbidity-relevant medication accounted for (a) ‘drugs against heart insufficiency’ (digitalis glycosides, diuretics, anti-arrhythmic drugs); (b) ‘drugs against cardiovascular diseases’ (statins, beta-blockers, acetylcholinesterase inhibitors [ACE inhibitors], angiotensin-I-antagonists); (c) ‘anti-acid drugs’ (proton-pump inhibitors [PPI], histamine-H2 blockers); (d) ‘anti-clotting medication’ (antiplatelet drugs, heparin (−derivates), vitamin-K antagonists – but no novel oral anti-coagulant drugs [NOACs] owing to their low prescription rate [413 patients, 1.1%]); (e) ‘anti-diabetic agents’; (f) ‘anti-depressants’; (g) ‘drugs for obstructive airway disease’ (long-acting beta2 agonists [LABA], long-acting muscarinic antagonists [LAMA], inhalative corticosteroids [ICS] and their combinations); and (h) ‘drugs for treatment of pulmonary hypertension’ (sildenafil, prostaglandin, bosentan, ambrisentan, sitaxentan, tadalafil, further referred to as specific PH drugs). The comorbidity-relevant medication was assessed based on the Anatomical Therapeutic Chemical (ATC) codes of substances prescribed during QDiag. For details, see Table 2.

Patients were assumed to be treated if a diagnosis was combined with at least one prescription for any therapeutic substance using the following combinations: (a) congestive heart failure: beta-blockers, ACE inhibitors, angiotensin-I-antagonists, digitalis glycosides, diuretic drugs; (b) cardiac arrhythmia: digitalis glycosides, anti-arrhythmic drugs, beta-blockers; (c) IHD: statins, beta-blockers, ACE inhibitors, angiotensin-I-antagonists, antiplatelet drugs, vitamin-K antagonists, heparin (−derivates); (d) valvular disease: beta-blockers, ACE inhibitors, digitalis glycosides; (e) hypertension: beta-blockers, ACE inhibitors, angiotensin-I-antagonists, diuretic drugs; (f) peripheral vascular disorders: antiplatelet drugs, vitamin-K antagonists, heparin (−derivates), statins; (g) diabetes: anti-diabetic agents; (h) GERD: PPI, histamine-H2 blockers; (i) COPD: LABA, LAMA, ICS or combinations; (j) PH: specific PH drugs, diuretics; and (k) depression: anti-depressants.

### Statistical analysis

For the complete ILD cohort as well as stratified by subtype, we assessed baseline characteristics, comorbid conditions, and medication profiles in a descriptive analysis.

Subsequently, we identified associations between comorbidity, treatment pattern, and survival via multivariate Cox models [29] with age, gender, and subtype as pre-fixed covariates. Comorbid conditions were classified as ‘treated’ and ‘untreated’ depending on the patients’ medication profiles. These findings were visualized in the form of a ‘comorbidome’, which combines information on hazard ratios (HRs) and comorbidity prevalence [8, 30]. A drug-extended Cox model including the pharmaceutical substances prescribed was performed as secondary analysis. Comorbid conditions respectively pharmaceutical substances considered in this selection process are further referred to as impact factors.

For variable selection, we included all conditions with a prevalence of at least 5.0% and relied on the LASSO method [31, 32], which can manage collinear input variables quite efficiently [33]. To calculate HRs, 95% confidence intervals (CI), and *p*-values, which are not part of LASSO itself, we ran ‘traditional’ Cox models with the covariates identified by LASSO. This procedure has been accepted to address issues of multiple testing adequately [34].

To check the robustness of our results, we performed two sensitivity analyses (SA). Considering the chronic nature of the comorbid conditions, we extended the observation timeframe for SA1 to the four quarters before QDiag. Moreover, we required a minimum of two outpatient diagnoses within two different quarters or at least one inpatient diagnosis. For drug prescriptions, a minimum of two prescriptions within two different quarters during the respective timeframe was required. For SA2, the four quarters from QDiag until three quarters after QDiag were screened for confirmed outpatient and inpatient ILD diagnoses. Only patients with a minimum of two diagnoses within at least two quarters were included. If a patient died during the prospective observation period, ILD diagnoses were required in all quarters observed with a maximum of one quarter without an ILD diagnosis. All analyses were performed at a significance level of 5% using the software packages SAS, version 9.3, and R, version 3.3.3. Comorbidomes were constructed using Microsoft Excel, version 2010.

## Results

### Population characteristics

Among the 36,821 patients identified, 13,985 received their diagnosis by a pulmonologist, 20 patients who had CTD were diagnosed by a rheumatologist, and 15,731 had inpatient diagnoses. Another 6338 individuals received both outpatient specialist and inpatient diagnoses. Seven hundred forty-seven patients were diagnosed by an internal specialist only.

The complete ILD cohort predominantly consisted of individuals with IIP (*n* = 14,453; 39.3%), sarcoidosis (*n* = 9106; 24.7%), and other fibrosing ILDs (*n* = 7187; 19.5%), while all other ILD-subtypes combined accounted for 6075 individuals (16.5%). The mean age at diagnosis was 66.0 years and 29,704 ILD-patients were male. The number of comorbid conditions ranged between 0 (4.4% of the population) and 18. Median survival was 29.0 months with a death rate of 31.0% (Table 1). Higher comorbidity burden was associated with increased mortality (Additional file 1: Figure S1).

**Table 1:** Baseline characteristics of the study sample

Study sample		
N	36,817	%
Idiopathic interstitial pneumonias (IIP)	14,453	39.3
Other fibrosing ILDs (OFI)	7187	19.5
Sarcoidosis (SARC)	9106	24.7
Drug-associated ILDs (DAI)	407	1.1
Pneumoconiosis (PNE)	1579	4.3
Radiation-associated pneumonitis (RAP)	464	1.3
Eosinophilic pneumonia (EPP)	1518	4.1
Hypersensitivity pneumonitis (HP)	967	2.6
Connective tissue disease-associated ILD (CTD)	1140	3.1
Ø age (years) at diagnosis (SD)	66.0	14.6
Male gender	20,704	56.2
Ø survival in months (SD)	29.6	0.31
Dead at end of observation period	11,422	31.0
Median number of comorbidities (IQR)^a^	5.0	[3.7]
Non-ILD-related hospitalization (%) during observation	24,665	67.0
ILD-related hospitalization (%) during observation	3304	9.0

All figures as N (%) unless reported otherwise

*IQR*, interquartile range, *SD* standard deviation

^a^31 comorbid conditions from the Elixhauser Index amended by PH, lung cancer, GERD, IHD, thrombosis, and OSAS yields a maximum of 37

Information on subtype-specific characteristics can be found in Additional file 2: Table S1.

### Comorbidity profile and associated medication

Having a prevalence below 5% excluded ‘pulmonary circulation disorders (excluding PH)’, ‘paralysis’, ‘other neurological disorders’, ‘peptic ulcer disease’, ‘AIDS/HIV’, ‘lymphoma’, ‘blood loss anaemia’, ‘alcohol abuse’, ‘drug abuse’, ‘psychoses’, and ‘thromboembolism’.

**Fig. 2:**
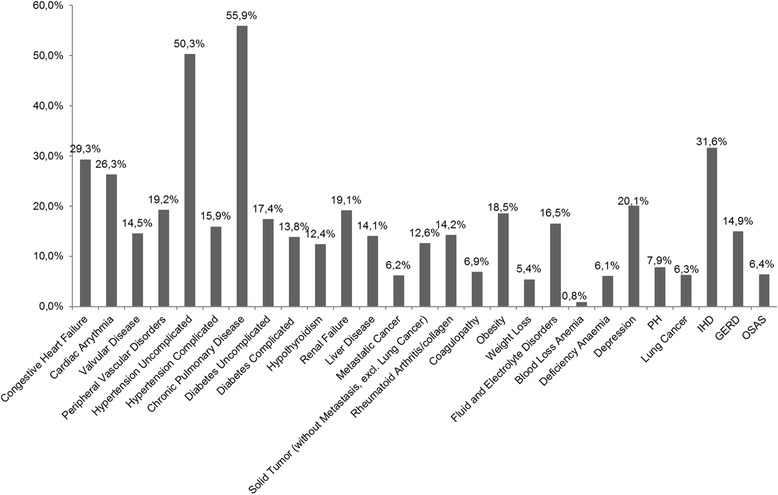
Comorbidity profile for the total cohort accounting for conditions with a prevalence ≥5.0%

More than half the patients had ‘COPD’ or ‘uncomplicated hypertension’ and almost one third had IHD (Fig. 2). These conditions were also the most prevalent in the distinct subtypes. ‘Metastatic cancer’, ‘coagulopathy’, and ‘deficiency anaemia’ were of low prevalence, whereas the ILD-relevant conditions ‘lung cancer’, ‘PH’, and ‘OSAS’ were observed in 6.3%, 7.9%, and 6.4% of the patients, respectively. The comorbidity profiles observed for distinct ILD subtypes resembled those within the total cohort except for a substantially higher prevalence of ‘metastatic cancer’ and ‘solid tumours’ in radiation-associated pneumonitis and of ‘rheumatoid arthritis/connective tissue disorder in CTD (Additional file 3: Table S2).

Prescription rates of comorbidity-relevant drugs (Table 2) generally ranged below the corresponding comorbidity prevalence. For 6 out of 13 comorbid conditions, more than one third of the patients did not receive any related drug treatment. ‘Lack of treatment’ was particularly pronounced for ‘depression’, ‘diabetes without complications’, and ‘COPD’, for which only a minority of patients received comorbidity-relevant drugs (35.8%, 39.1%, 46.0% respectively). In contrast, almost all patients with cardiac or cardiovascular diseases had related drug prescriptions. However, the prescription rates for the distinct substances at disposal varied substantially (Table 3). This general trend was largely transferable to the distinct subtypes (Additional file 4: Table S3).

**Table 2:** Prescription patterns of comorbidity-relevant medication at baseline

Individuals with prescriptions of therapeutic agents for comorbid conditions		
	N	%
Treatment of heart insufficiency/cardiac arrhythmia	11,267	30.6
Digitalis glycosides (ATC code C01AA)	1516	4.1
Anti-arrhythmic drugs (ATC code C01BD)	541	1.5
Diuretic drugs (ATC code C03)	10,631	28.8
Treatment of cardiovascular disease	19,376	52.6
Statins (ATC code C10AA)	5881	16.0
Beta-blockers (ATC code C07)	11,038	30.0
ACE inhibitors (ATC codes C09AA C09B)	9309	25.3
Antiotensin-I-antagonists (ATC codes C09CA C09D)	4664	12.7
Treatment with anti-clotting medication	7692	20.9
Antiplatelet drugs (ATC code B01AC)	4071	11.1
Vitamin-K antagonists (ATC code B01AB)	2428	6.60
Heparin (−derivates) (ATC code B01AA)	2285	6.2
Treatment with anti-acid drugs	14,596	39.6
Proton-pump inhibitors (PPI) (ATC code A02BC)	14,171	38.5
Histamine-H2 blockers (ATC code A02BA)	463	1.3
Treatment with anti-depressants (ATC code N06A)	3952	10.7
Treatment with anti-diabetic drugs (ATC codes A10A, A10B)	5891	16.0
Treatment with drugs against obstructive airway disease	11,021	29.9
Long-acting beta2 agonists (LABA) (ATC code R03AC)	2640	7.2
Long-acting muscarinic antagonists (LAMA) (ATC code R03BB)	4690	12.7
Inhaled corticosteroids (ICS) (ATC code R03BA)	2794	7.6
Combination product LABA/ICS (ATC code R03AK)	5135	13.9
Combination product LABA/LAMA (ATC code R03AL)	40	0.1
Treatment with specific PH drugs (ATC codes G04BE03, B01AC09, C03KX)	265	0.7

**Table 3:** Proportion of treated comorbid conditions

Share of diagnosed individuals with comorbidity-relevant prescription at baseline			
	N diagnosed	N treated	% treated
Congestive heart failure (EI group 1)	10,793	9514	88.1
Beta-blockers		5093	53.5
ACE inhibitors		3995	42.0
Angiotensin-I-antagonists		1752	18.4
Digitalis glycosides		1038	10.9
Diuretic drugs		5114	53.8
Cardiac arrhythmia (EI group 2)	9694	5382	55.5
Beta-blockers		4769	88.6
Digitalis glycosides		1256	23.3
Anti-arrhythmic drugs		512	9.5
Valvular disease (EI group 3)	5345	3957	74.0
Beta-blockers		2536	64.1
ACE inhibitors		1806	45.6
Diuretic drugs		2691	68.0
IHD	11,632	9357	80.4
Statins		3761	40.2
Beta-blockers		5730	61.2
ACE inhibitors		4292	45.9
Angiotensin-I-antagonists		1971	21.1
Antiplatelet drugs		2986	31.9
Heparin (−derivates)		1157	12.4
Vitamin-K antagonists		1187	12.7
Hypertension without complications (EI group 6)	19,059	17,594	92.3
Beta-blockers		7255	41.2
ACE inhibitors		6433	36.6
Angiotensin-I-antagonists		3241	18.4
Diuretic drugs		4936	28.1
Hypertension with complications (EI group 7)	5866	5301	90.4
Beta-blockers		2813	53.1
ACE inhibitors		2221	41.9
Angiotensin-I-antagonists		1222	23.1
Diuretic drugs		2427	45.8
PH	4537	1771	39.0
PAH drugs		237	13.4
Diuretic drugs		1699	95.9
COPD (EI group 10)	20,595	9473	46.0
LABA		2431	25.7
LAMA		4376	46.2
ICS		2138	22.6
Peripheral vascular disorders (EI group 4)	6692	3391	50.7
Antiplatelet drugs		1566	46.2
Heparin (−derivates)		698	20.6
Vitamin-K antagonists		581	17.1
Statins		1928	56.9
Diabetes without complications (EI group 11)	6403	2505	39.1
Diabetes with complications (EI group 12)	5025	3337	66.4
Depression (EI group 31)	7410	2656	35.8
GERD	5594	3629	64.9
PPI		3545	97.7
Histamin-H2 antagonists	5594	99	2.7

percentages at substance level refer to the treated population

*EI* Elixhauser Comorbidity Index

### Associations between clinical characteristics and mortality

**Fig. 3:**
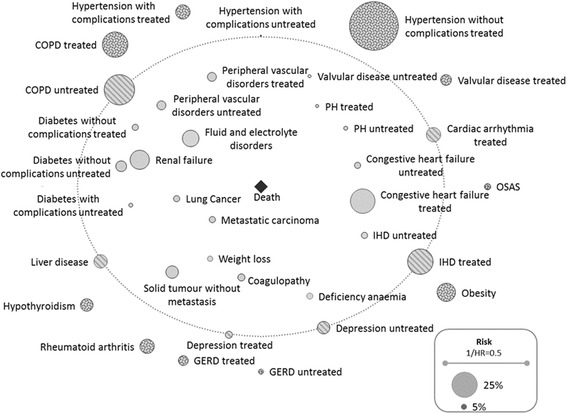
ILD comorbidome based on results of the LASSO selection for the comorbidity-only Cox model

The comorbidity-only Cox model (columns two and three of Table 4) and the drug-extended Cox model (columns four and five of Table 4) both revealed a significantly positive association of male gender, age, and non-sarcoidosis ILDs with mortality. Both models yielded similar HRs, whereupon the drug-extended model in general exhibited slightly less pronounced associations. The mortality risk was generally higher in untreated comorbid conditions than in treated ones. Most conditions were negatively associated with survival, but positive associations were observed for ‘OSAS’, ‘treated valvular disease’, ‘treated hypertension with complications’, ‘obesity’, ‘hypothyroidism’, ‘treated GERD’, and ‘rheumatoid arthritis/connective tissue disorder’ in both Cox models. For the comorbidity-only Cox model, corresponding findings are visualized as a comorbidome (Fig. 3). Referring to the drug-extended Cox model ‘Statins’, ‘beta-blockers’, ‘ACE inhibitors’, ‘angiotensin-I-antagonists’, ‘anti-arrhythmic drugs’, ‘heparin (−derivates)’ and ‘ICS’ showed an independent positive association, whereas ‘diuretics’, ‘antiplatelet drugs’, ‘PPIs’, ‘H2-antagonists’, ‘specific PH drugs’, and ‘anti-depressants’ demonstrated a negative association with survival.

**Table 4:** Association of comorbid conditions and respective treatments with survival according to Cox proportional hazard regression models

Variable		Comorbidity-only model		Drug-extended model	
		HR	95% CI	HR	95% CI
Comorbidity profile	Congestive heart failure treated (EI group 1)	1.53^***^	[1.46;1.60]	1.50^***^	[1.43;1.58]
	Congestive heart failure untreated (EI group 1)	1.58^**^	[1.42;1.76]	1.53^***^	[1.39;1.68]
	Cardiac arrhythmia treated (EI group 2)	1.03^n.s.^	[0.98;1.08]	Not selected	
	Cardiac arrhythmia untreated (EI group 2)	Not selected		1.02^ns^	[0.95;1.08]
	Valvular disease treated (EI group 3)	0.93^*^	[0.88;0.99]	0.95	[0.89;1.00]
	Valvular disease untreated (EI group 3)	1.10^*^	[1.01;1.21]	1.13^**.^	[1.03;1.24]
	Pulmonary hypertension treated	1.40^***^	[1.31;1.56]	1.22^***^	[1.13;1.31]
	Pulmonary hypertension untreated	1.43^***^	[1.31;1.56]	1.55^***^	[1.42;1.70]
	Peripheral vascular disorders treated (EI group 5)	1.11^***^	[1.05;1.18]	1.12^***^	[1.05;1.18]
	Peripheral vascular disorders untreated (EI group 5)	1.13^***^	[1.07;1.20]	1.13^***^	[1.07;1.20]
	Hypertension without complications treated (EI group 6)	0.80^**^	[0.73;0.88]	Not selected	
	Hypertension without complications untreated (EI group 6)	Not selected		1.00	[0.94;1.05]
	Hypertension with complications treated (EI group 7)	0.86^***^	[0.82;0.90]	0.89^**^	[0.83;0.95]
	Hypertension with complications untreated (EI group 7)	1.01^ns^	[0.88;1.16]	0.86^***^	[0.75;0.97]
	COPD treated (EI group 10)	0.91^***^	[0.86;0.95]	0.98^ns^	[0.94;1.03]
	COPD untreated (EI group 10)	0.97^ns.^	[0.92;1.01]	Not selected	
	Diabetes without complications treated (EI group 11)	1.09^*^	[1.02;1.17]	Not selected	
	Diabetes without complications untreated (EI group 11)	1.16^**^	[1.10;1.22]	0.99^ns^	[0.60;1.62]
	Diabetes with complications treated (EI group 12)	1.12^***^	[1.06;1.20]	1.03^ns^	[0.63;1.59]
	Diabetes with complications untreated (EI group 12)	1.20^***^	[1.12;1.30]	1.19^***^	[1.11;1.28]
	Hypothyroidism (EI group 13)	0.89^***^	[0.84;0.95]	0.90^***^	[0.85;0.95]
	Renal failure (EI group 14)	1.27^***^	[1.21;1.33]	1.26^***^	[1.20;1.32]
	Liver disease (EI group 15)	1.05^ns.^	[1.00;1.11]	1.04^ns^	[0.99;1.10]
	Metastatic carcinoma (EI group 19)	2.51^***^	[2.35;2.67]	2.51^***^	[2.36;2.68]
	Solid tumour without metastasis (EI group 20)	1.15^***^	[1.10;1.21]	1.15^***^	[1.10;1.21]
	Rheumatoid Arthritis/Connective Tissue Disorder (EI group 21)	0.89^***^	[0.84;0.95]	0.88^***^	[0.83;0.94]
	Coagulopathy (EI group 22)	1.40^***^	[1.32;1.49]	1.43^***^	[1.35;1.52]
	Obesity (EI group 23)	0.87^***^	[0.83;0.92	0.90^***^	[0.83;0.94]
	Weight loss (EI group 24)	1.56^***^	[1.46;1.66]	1.53^***^	[1.44;1.63]
	Fluid and electrolyte disorders (EI group 25)	1.74^**^	[1.67;1.82]	1.70^***^	[1.63;1.78]
	Deficiency anaemia (EI group 27)	1.11^**^	[1.04;1.19]	1.10^**^	[1.03;1.18]
	Depression treated (EI group 31)	1.07^ns^	[1.00;1.15]	1.00^ns^	[0.95;1.06]
	Depression untreated (EI group 31)	0.99^ns.^	[0.93;1.04]	Not selected	
	IHD treated	0.97^ns^	[0.93;1.01]	Not selected	
	IHD untreated	1.33^***^	[1.24;1.42]	1.04^***^	[0.98;1.09]
	GERD treated	0.92^**^	[0.87;0.98]	0.89^***^	[0.83;0.95]
	GERD untreated	0.92^*.^	[0.84;0.99]	0.95^ns^	[0.87;1.03]
	OSAS	0.76^***^	[0.70;0.82]	0.77^***^	[0.71;0.84]
	Lung cancer	1.86^***^	[1.74;1.98]	1.80^***^	[1.69;1.92]
Medication intake	Digitalis glycosides			1.07^ns^	[0.99;1.16]
	Anti-arrhythmic drugs			0.86^*^	[0.75;0.98]
	Diuretic drugs			1.24^***^	[1.18;1.31]
	Statins			0.85^***^	[0.80;0.90]
	Beta-blockers			0.92^***^	[0.88;0.97]
	ACE inhibitors			0.83^***^	[0.79;0.87]
	Angiotensin-I-antagonists			0.73^***^	[0.68;0.78]
	Antiplatelet drugs			1.08^*^	[1.02;1.15]
	Heparin (−derivates)			0.84^***^	[0.98;1.11]
	Vitamin-K antagonists			1.04^ns^	[0.78;0.90]
	Proton pump inhibitors			1.05^*^	[1.00;1.09]
	H2-antagonists			1.24^**^	[1.07;1.43]
	Treatment with anti-depressants			1.12^***^	[1.05;1.18
	Treatment with anti-diabetic drugs			1.07^ns^	[0.66;1.75]
	Long-acting beta2 agonists (LABA)			0.98^ns^	[0.91;1.05]
	Long-acting muscarinic antagonists (LAMA)			1.05^ns^	[1.00;1.11]
	Inhaled corticosteroids (ICS)			0.89^**^	[0.82;0.97]
	Combination product LABA/ICS			not selected	
	Combination product LABA/LAMA			not selected	
	Specific PH drugs			1.85^***^	[1.56;2.18]

All figures adjusted for age, gender, and ILD subtype

Comorbidity-only Cox model: concordance = 0.790, R^2^ = 0.286; AIC = 219,409.6

Drug-extended Cox model: concordance = 0.794, R^2^ = 0.2904; AIC = 219,215.4

*HR* hazard ratio, *CI* confidence interval, *EI* Elixhauser Comorbidity Index

Significance codes: *p* < 0.001 ‘^***^’ | *p* < 0.01 ‘^**^’ | *p* < 0.05 ‘^*^’ | not significant ^‘ns’.^ ‚ not selected: disregarded by LASSO selection

**Fig. 4:**
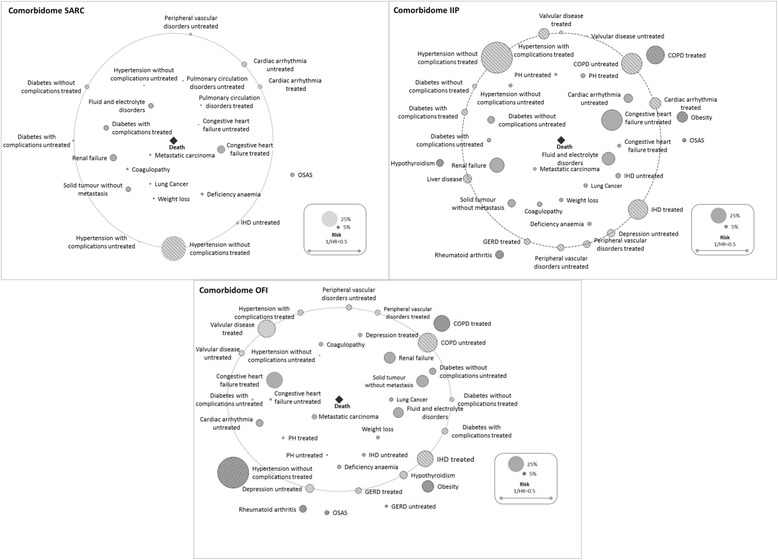
IIP, other fibrosing ILDs- and sarcoidosis-specific comorbidomes based on results of the LASSO selection for the comorbidity-only Cox model

Within the analyses stratified by subtype (Additional file 4: Table S3), all significant effects mirrored the total cohort, yet the pre-selected impact factors and the judgement of their significance differed substantially by ILD subtypes. Additionally, we found no positive association between any comorbid condition and survival for drug-associated ILD, radiation-associated pneumonitis, and HP, and only a single positive association for sarcoidosis (‘OSAS’), pneumoconiosis (‘OSAS’), and CTD (‘treated valvular disease’). Likewise, we identified just two positive associations for eosinophilic pneumonia (‘treated hypertension with complications’, ‘treated IHD’). However, we discovered five positive associations for IIP (‘treated COPD’, ‘hypothyroidism’, ‘rheumatoid arthritis/connective tissue disorder’, ‘obesity’, and ‘OSAS’) and six for other fibrosing ILDs (‘treated hypertension without complications’, ‘treated COPD’, ‘rheumatoid arthritis/connective tissue disorder, ‘obesity’, ‘untreated GERD’, and ‘OSAS’). Figure 4 summarizes the corresponding results for IIP, other fibrosing ILDs, and sarcoidosis, whilst the comorbidomes for the remaining ILD-subtypes are represented as Additional file 5: Figure S3.

The drug-extended models indicated an independent advantageous impact of treatment with statins for IIP, other fibrosing ILDs, sarcoidosis, pneumoconiosis, and eosonophilic pneumonia and of treatment with heparin (−derivates) for IIP, other fibrosing ILDs, sarcoidosis, and pneumoconiosis. In contrast to the total cohort analysis, antiplatelet drugs had no detrimental association with survival in any subtype, whereas vitamin-K antagonists were associated with increased mortality in radiation-associated pneumonitis. Moreover, an increased mortality risk was observed for patients with other fibrosing ILDs treated with histamine-H2 antagonists and for sarcoidosis patients treated with PPIs; however, the PPI treatment was associated with a survival benefit among CTD patients (Additional file 6: Table S4).

### Sensitivity analyses

Details on the SAs are found within Additional files 6, 7, 8, and 9: Tables S4–S7 and Additional file 10: Figure S2.

#### Sensitivity Analyses 1 (SA1)

For all conditions except for ‘COPD’ and ‘fluid and electrolyte disorders’ prevalence rates were similar to the main analysis. Prescription rates of comorbidity-relevant drugs were reduced particularly for statins and COPD-related drugs.

Comparing the comorbidity-only Cox model to the main analysis, ‘treated COPD’ and ‘treated diabetes with complications’ lost their significance, whereas ‘liver disease’ gained significance. For the drug-extended Cox model, the exclusion of ‘lung cancer’ during the LASSO selection process and a highly significant positive association of ‘treated diabetes without complications’ with survival were the only relevant differences regarding comorbid conditions. Moreover, referring to comorbidity-relevant drugs, the beneficial impact of heparin-(derivates) observed in the main analysis could not be replicated.

#### Sensitivity Analyses 2 (SA2)

Twenty-one thousand five hundred eighty-one individuals were considered for the SA2, but neither the age and gender distribution nor the median observation period for this population differed from the main analysis. Comorbidity prevalence differed only marginally but the sample presented lower proportions of comorbidity treatment. In contrast to the main analysis, ‘treated diabetes without complications’ and ‘untreated hypertension with complications’ demonstrated a significant detrimental association in the comorbidity-only Cox model, whereas ‘treated hypertension without complications’ and ‘hypothyroidism’ lost their beneficial effect. Moreover, the negative impact of ‘treated diabetes with complications’ could not be transferred from the main analysis to SA2. Regarding the drug-extended Cox model highly significant positive associations of treated as well as untreated COPD were the only noteworthy differences from the main analysis.

## Discussion

Our study emphasizes the relevance of comorbidities and their consequent treatment across various ILDs. The results obtained through this study suggest the need for improving comprehensive pharmaceutical comorbidity management in ILDs, with a particular emphasis on the treatment of ILD-relevant comorbid conditions such as COPD (54.0% untreated) and GERD (35.1% untreated). Moreover, we perceived ILD subtype-specific comorbidity profiles. In this regard, the current ICD-10 coding system needs to be acknowledged as not being sufficiently differentiated to precisely separate distinct fibrosing ILDs from one another. Therefore, patients in the IIP and the other fibrosing ILDs group may be substantially more heterogeneous than in the other subtypes. Although within less frequent subtypes sample size issues might explain the observed differences, the LASSO method, which selects conditions based on relevance and not just on significance, provided first insights into subtype-specific comorbidity management approaches.

As expected, highly prevalent, potentially age-associated comorbid conditions were not necessarily those with the strongest impact on survival [35–37]. Yet generally, we observed a clinically meaningful association with a higher mortality risk in the case of untreated distinct comorbidities. This emphasizes the need for active screening for and treatment of comorbidity in distinct ILDs. This might be highlighted by using the example of PH, which is recognised as a marker of diseases progression and prognosis in ILDs. PH frequently complicated the course of many ILDs depicted in our data and was identified as a crucial impact factor for all subtypes but radiation-associated pneumonitis. However, the advantage of specific PH drugs remains unclear. Recent trials have not only failed to demonstrate a benefit of PH-drugs but even reported potential detrimental effects in IPF [38]. Thus, treatment options are limited. Our data show that most patients are treated according to clinical practice with diuretics (96% of treated PH patients), while the use of PH drugs is low (14% of treated PH patients). Only in the subgroup of CTD-ILD and PH did 57% of the treated patients receive specific PH drugs that complied with current guidelines [39].

‘COPD’, ‘treated hypertension’, rheumatoid arthritis/connective tissue disorders’, and ‘OSAS’ were positively associated with survival in the complete cohort, but this was generally not the case on the level of the distinct ILD strata. Observations regarding ‘COPD’ and ‘treated hypertension’ might be explained by an early detection bias. This means that individuals with a known chronic condition are under frequent medical control, which increases the likelihood of earlier ILD diagnosis or that symptoms related to the comorbid condition yield an earlier detection of the ILD. In part, this explanation also applies to ‘rheumatoid arthritis/connective tissue disorders’ that can manifest as CTD-ILDs. Yet from a clinical point of view, this comorbidity should not occur in other subtypes. In contrast, a recent study concerning OSAS reports that CPAP therapy enhances the capability of IPF patients to perform activities of daily living, and the authors also proposed a mitigating effect on mortality [40], which corresponds with our own observation on the general impact of OSAS.

In addition to investigating the associations of comorbidity prevalence and its treatment to survival, we analysed the independent impact of distinct comorbidity-related drugs. Currently, the role of anti-acid treatment is particularly under discussion [7, 8, 41, 42]. As reported before for IPF [7], we could not find an association between anti-acid treatment and improved survival among the distinct ILDs, yet we simultaneously observed an independent negative impact of anti-acid drugs in other fibrosing ILDs and sarcoidosis, as previously shown for IPF [8]. Reverse causality must be considered as an explanatory factor to solve this apparent contradiction.

At the subtype level, we found no association between antiplatelet drugs and survival [8]. Yet, there was a positive impact of heparin (−derivates) in IIP, other fibrosing ILDs, sarcoidosis, and pneumoconiosis, but vitamin-K antagonists had no significant impact on survival in any subtype (except for radiation-associated pneumonitis). This needs to be further investigated, as recent analyses report a detrimental association between anticoagulants and survival or disease progression [20, 43, 44]. These studies focus on warfarin and IPF, whereas we studied all forms of vitamin-K antagonists and heparins within several ILD subtypes. Apparently, the role of anticoagulants is not entirely understood and a detailed examination of different drug entities is urgently required, especially for subtypes other than IPF. Here, particularly the role of NOACs – which were not included in our analyses as only 413 (1.1%) patients received corresponding prescriptions – should be further investigated.

Although our observed positive impact of drugs for the treatment of cardiovascular diseases supports recent studies claiming benefits for statin therapy [21, 45], the results of our analysis should be cautiously interpreted. Different patient groups and methodological approaches hamper a sound between-study comparison and our research focuses on a claims data-based study [46].

Compared with studies from specialized ILD centres [7, 8], our study displayed more frequent age-associated comorbidities (e.g., arterial hypertension, IHD, diabetes). Possibly, this display represents an incorporation of diagnoses from primary, secondary, and tertiary care, where priority setting for distinct conditions is assumed to be different. Additionally, different patient cohorts introduce substantial variation in regards to comorbidity prevalence [16]. Thus, direct comparisons are a sensitive issue. Moreover, the comorbidity burden was assessed based on ICD-10 codes, which could produce potential coding errors [47]. Furthermore, the coding practice may be triggered to some extent by remuneration relevance. One example could be the observed high prevalence of COPD, for which additional payments exist in the form of disease management programme bonuses. To ensure sound comorbidity diagnosis, we restricted our analysis to confirmed diagnoses and required the multiple documentation of comorbidity diagnoses within SA1. Given the high concordance between the main analysis and SA1, we assume that the risk of false-positive comorbidity diagnoses is of subordinated relevance.

Currently, the ICD coding system does not allow differentiation between different IIPs and other fibrosing ILDs. Since the documented codes could not be validated with medical files, the possibility exists that some of the patients may have been misclassified. To some extent, this possibility is supported by the unexpectedly high proportion of individuals with eosinophilic pneumonia as well as by the high share of individuals with comorbid 'rheumatoid arthritis/connective tissue disorders' in non-CTD-ILDs. Here, the assignment of individuals with polyarthropathy to other subtypes than CTD may result due to the coding requirement that CTD-ILD should only be diagnosed in the presence of systemic connective tissue disorders (ICD-10 codes M33-M335) but not in the case of polyarthropathy (ICD10-code M11-M14). Indeed, we observed a particularly high prevalence of polyarthropathy in the heterogeneous subtypes of other fibrosing ILDs and IIP and only within these two subtypes 'rheumatoid arthritis/connective tissue disorders' presented a significant association with survival. This suggests that the assignment to distinct ILD-subtypes is more in accordance with coding requirements than with a clinical perspective on aetiology, which might affect conclusions on the associations between comorbidity presence and mortality. This underscores that thorough differential diagnostic of patients with ILDs is essential to initiate appropriate ILD management.

To avoid using a misdiagnosis, we only allowed confirmed ILD diagnoses by medical specialists combined with related diagnostic procedures, applied specific code combinations (radiation-associated pneumonitis + diagnosis of malignacy, CTD + diagnosis of autoimmune disease), and, lastly, we disregarded patients diagnosed with multiple forms of ILDs. Although misclassification of subtypes cannot be excluded, the results of the main analysis correlated well with those of SA2 that applied a prospective validation of the initial ILD diagnosis. Therefore, we are convinced that a misclassification of subtypes does not crucially affect the associations found between comorbidity and survival.

Due to the lack of information on clinical parameters from the claims data, we could not account for the severity of the ILDs or that of comorbidities [46]. However, not included in our data was the baseline information on lung function. Disease severity remains a major impact factor on survival in ILDs. Therefore, this baseline information on lung function should be considered as an effect modificator because FVC and DLCO are established predictors for survival, e.g. in IPF [48] or SSc-related ILD [49]. Additional knowledge lacking from the claims data were reliable ICD codes for lifestyle factors such as smoking, dietary habits, and physical activity. These factors could not be incorporated into the analyses.

Moreover, the decision to pharmaceutically treat a comorbid condition depends on many factors such as the medical need for treatment, the potential interactions of treatment regimens, the patients’ individual performance status, and patient/physician preferences. Without the connection to medical files, the aspect of confounding by indication is clearly acknowledged to avoid potential misconclusions on treatment recommendations. However, even a connection with medical files cannot eliminate issues of adherence, meaning that the prescription of a distinct drug is not necessarily linked to the medication intake by the patient.

Likewise, we limited our research to baseline comorbidity only and did not incorporate newly occurring conditions in the course of the ILD. By this, further relevant conditions may have been disregarded. For example, Hyldgaard et al. have shown that substantial comorbidities are diagnosed after IPF-diagnosis but they only observed an additional mortality risk for late onset of cardiovascular diseases and not for other conditions [7]. Hence, baseline comorbidity restriction may presumably only affect the magnitude of HRs but not general statements on significance of the comorbidities reported.

 Last, the nature of this analysis does not allow a distinction between ILD-related and non-ILD-related mortality. Indeed, all-cause mortality might not reflect the impact of distinct comorbid conditions on ILD-related mortality comprehensively. In particular, the effects of conditions that present substantial disease-specific lethality might be overestimated. Given recent evidence on an enhanced mortality effect of combined ILD and lung cancer [12, 50], it seems justified to assume corresponding interactions for other conditions as well.

In contrast, claims data remain less prone to selection bias than primary data-based cohorts. Thus, a realistic picture of comorbidity burden and treatment practice in daily routine care can be assumed. Moreover, large sample sizes enable analysis of the comorbidity profiles of rare ILD subtypes for which reference literature is sparse.

For enhanced ILD-management thorough comorbidity assessment seems paramount: We based our assessment on the Elixhauser Index which was amended by ILD-relevant comorbidities (‘GERD’, ‘OSAS’, ‘PH’, and ‘lung cancer’). Other than the often applied Carlson Index [36], the Elixhauser Index more stringently addresses chronic conditions related to high morbidity but not necessarily to high mortality [26]. Thus, our approach seems superior in the field of ILDs, which are quite heterogeneous regarding their survival prognosis.

To further alleviate management decisions, we finally disentangled untreated and treated comorbidity in regards to comorbidity-relevant drugs. In our opinion, this approach appears to be preferable to a simple prevalence-based analysis (comorbidity yes/no) that might fail to detect the peculiar relevance of pharmaceutical comorbidity management.

## Conclusion

In conclusion, improved management of comorbidities in ILDs should address all survival-relevant conditions and not be limited to risk factors and sequelae. Consequently, more stringent pharmaceutical treatment seems to be advisable because our analyses provide first insights that untreated comorbidity tends to affect survival in the various ILDs more detrimentally than treated comorbidity. However, since insurance claims data do not address clinical characteristics of ILD patients comprehensively, further analyses of clinical cohorts with more differentiated information on ILD subtype and disease severity is needed. To improve patient benefit meaningfully, detailed longitudinal investigations of the mutual interactions between comorbidity and its treatment at the substance level should be encouraged. In parallel, a stronger emphasis could be dedicated to evaluating the benefits of non-pharmacological disease management.

## Additional files

Additional file 1: **Figure S1.** Kaplan–Meier plots according to comorbidity quartile. (DOC 35 kb)

Additional file 2: **Table S1.** Baseline characteristics for the population stratified by ILD subtype. (DOC 81 kb)

Additional file 3: **Table S2.** Prescription patterns of comorbidity-relevant medication at the subtype level. (DOC 75 kb)

Additional file 4: **Table S3.** ILD subtype-specific hazard ratios as per the comorbidity-only Cox model. (DOC 97 kb)

Additional file 5: **Figure S3.** Pneumoconios-, Drug-associated ILD-, Radiation-asscociated pneumonitis-, Eosinophilic pneumonia-, HP- and CTD-specific comorbidomes based on results of the LASSO selection for the comorbidity-only Cox model. (DOC 648 kb)

Additional file 6: **Table S4.** Baseline characteristics of the study sample within Sensitivity Analysis 2. (DOC 56 kb)

Additional file 7: **Table S5.** Prescription patterns of comorbidity-relevant medication according to sensitivity analyses. (DOC 74 kb)

Additional file 8: **Table S6.** Proportion of treated comorbid conditions according to sensitivity analyses. (DOC 60 kb)

Additional file 9: **Table S7.** Associations of clinical characteristics and survival within the sensitivity analyses. (DOC 146 kb)

Additional file 10: **Figure S2.** Comorbidity prevalence in main analysis compared with sensitivity analyses. (DOC 104 kb)

## Abbreviations

ACE: Acetylcholinesterase
AIC: Akaike information criterion
ATC: Anatomical therapeutic chemical code
CI: Confidence interval
COPD: Chronic obstructive pulmonary disease
CT: Computerized tomography
CTD: Connective tissue-associated ILD
DAI: Drug-associated ILD
DUA: Data use agreement
DZL: Deutsches Zentrum für Lungenforschung/German Center for Lung Research
EI: Elixhauser Index
EPP: Eosinophilic pneumonia
GERD: Gastro-esophageal reflux disease
HR: Hazard ratio
ICD: International statistical classification of diseases and related health problems
ICS: Inhalative corticosteroid
IHD: Ischaemic heart disease
IIP: Idiopathic interstitial pneumonia
ILD: Interstitial lung disease
IPF: Idiopathic pulmonary fibrosis
IQR: Interquartile range
LABA: Long-acting beta2 agonist
LASSO: Least absolute shrinkage and selction operator
NOAC: Novel anticoagulant drug
ns: Not significant
OFI: Other fibrosing ILDs
OSAS: Obstructive sleeping apnoea syndrome
PH: Pulmonary hypertension
PNE: Pneumoconiosis
PPI: Proton pump inhibitor
QDiag: Quarter of ILD diagnosis
RAP: Radiation-associated pneumonitis
SA: Sensitivity analysis
SARC: Sarcoidosis
SD: Standard deviation
WIdO: Wissenschaftliches Institut der AOK/Scientific Institute of AOK Statutory Health Insurance Funds

## References

[1] GBD 2015 Mortality and Causes of Death Collaborators (2016). Global, regional, and national life expectancy, all-cause mortality, and cause-specific mortality for 249 causes of death, 1980-2015: a systematic analysis for the global burden of disease study 2015. Lancet.

[2] King TEJ, Bradford WZ, Castro-Bernardini S, Fagan EA, Glaspole I, Glassberg MK, Gorina E, Hopkins PM, Kardatzke D, Lancaster L (2014). A phase 3 trial of pirfenidone in patients with idiopathic pulmonary fibrosis. N Engl J Med.

[3] Richeldi L, du Bois RM, Raghu G, Azuma A, Brown KK, Costabel U, Cottin V, Flaherty KR, Hansell DM, Inoue Y (2014). Efficacy and safety of nintedanib in idiopathic pulmonary fibrosis. N Engl J Med.

[4] Kreuter M, Cottin V. The Yin and Yang of Idiopathic Pulmonary Fibrosis. Eur Respir J. 2017;49(2). 10.1183/13993003.02316-2016 28232416

[5] Putcha N, Drummond MB, Wise RA, Hansel NN (2015). Comorbidities and chronic obstructive pulmonary disease: prevalence, influence on outcomes, and management. Semin Respir Crit Care Med.

[6] Valderas JM, Starfield B, Sibbald B, Salisbury C, Roland M (2009). Defining comorbidity: implications for understanding health and health services. Ann Fam Med.

[7] Hyldgaard C, Hilberg O, Bendstrup E (2014). How does comorbidity influence survival in idiopathic pulmonary fibrosis?. Respir Med.

[8] Kreuter M, Ehlers-Tenenbaum S, Palmowski K, Bruhwyler J, Oltmanns U, Muley T, Heussel CP, Warth A, Kolb M, Herth FJ (2016). Impact of comorbidities on mortality in patients with idiopathic pulmonary fibrosis. PLoS One.

[9] Nowinski A, Puscinska E, Goljan A, Peradzynska J, Bednarek M, Korzybski D, Kaminski D, Stoklosa A, Czystowska M, Sliwinski P, Gorecka D (2017). The influence of comorbidities on mortality in sarcoidosis: a observational prospective cohort study. Clin Respir J.

[10] Hyldgaard C, Hilberg O, Pedersen AB, Ulrichsen SP, Lokke A, Bendstrup E, Ellingsen T (2017). A population-based cohort study of rheumatoid arthritis-associated interstitial lung disease: comorbidity and mortality. Ann Rheum Dis.

[11] Patel NM, Lederer DJ, Borczuk AC, Kawut SM (2007). Pulmonary hypertension in idiopathic pulmonary fibrosis. Chest.

[12] Tomassetti S, Gurioli C, Ryu JH, Decker PA, Ravaglia C, Tantalocco P, Buccioli M, Piciucchi S, Sverzellati N, Dubini A (2015). The impact of lung cancer on survival of idiopathic pulmonary fibrosis. Chest.

[13] Nathan SD, Basavaraj A, Reichner C, Shlobin OA, Ahmad S, Kiernan J, Burton N, Barnett SD (2010). Prevalence and impact of coronary artery disease in idiopathic pulmonary fibrosis. Respir Med.

[14] Lancaster LH, Mason WR, Parnell JA, Rice TW, Loyd JE, Milstone AP, Collard HR, Malow BA (2009). Obstructive sleep apnea is common in idiopathic pulmonary fibrosis. Chest.

[15] Margaritopoulos GA, Antoniou KM, Wells AU. Comorbidities in Interstitial Lung Diseases. Eur Respir Rev. 2017;26(143). 10.1183/16000617.0027-2016 PMC9488735 28049126

[16] Raghu G, Amatto VC, Behr J, Stowasser S (2015). Comorbidities in idiopathic pulmonary fibrosis patients: a systematic literature review. Eur Respir J.

[17] Buendia-Roldan I, Mejia M, Navarro C, Selman M (2017). Idiopathic pulmonary fibrosis: clinical behavior and aging associated comorbidities. Respir Med.

[18] King C, Nathan SD (2013). Identification and treatment of comorbidities in idiopathic pulmonary fibrosis and other fibrotic lung diseases. Curr Opin Pulm Med.

[19] Kreuter M, Wuyts W, Renzoni E, Koschel D, Maher TM, Kolb M, Weycker D, Spagnolo P, Kirchgaessler KU, Herth FJ, Costabel U (2016). Antacid therapy and disease outcomes in idiopathic pulmonary fibrosis: a pooled analysis. Lancet Respir Med.

[20] Kreuter M, Wijsenbeek MS, Vasakova M, Spagnolo P, Kolb M, Costabel U, Weycker D, Kirchgaessler KU, Maher TM (2016). Unfavourable effects of medically indicated oral anticoagulants on survival in idiopathic pulmonary fibrosis. Eur Respir J.

[21] Kreuter M, Bonella F, Maher TM, Costabel U, Spagnolo P, Weycker D, Kirchgaessler KU, Kolb M (2017). Effect of statins on disease-related outcomes in patients with idiopathic pulmonary fibrosis. Thorax.

[22] Jaunzeme J, Eberhard S, Geyer S (2013). Wie “repräsentativ” sind GKV-Daten?. Bundesgesundheitsbl Gesundheitsforsch Gesundheitsschutz.

[23] Swart E, Gothe H, Geyer S, Jaunzeme J, Maier B, Grobe TG, Ihle P (2015). Gute Praxis Sekundärdatenanalyse (GPS): Leitlinien und Empfehlungen. Gesundheitswesen.

[24] Esposito DB, Lanes S, Donneyong M, Holick CN, Lasky JA, Lederer D, Nathan SD, O'Quinn S, Parker J, Tran TN (2015). Idiopathic pulmonary fibrosis in United States automated claims. Incidence, prevalence, and algorithm validation. Am J Respir Crit Care Med.

[25] Walsh SL, Wells AU, Desai SR, Poletti V, Piciucchi S, Dubini A, Nunes H, Valeyre D, Brillet PY, Kambouchner M (2016). Multicentre evaluation of multidisciplinary team meeting agreement on diagnosis in diffuse parenchymal lung disease: a case-cohort study. Lancet Respir Med.

[26] Elixhauser A, Steiner C, Harris DR, Coffey RM (1998). Comorbidity measures for use with administrative data. Med Care.

[27] Quan H, Sundararajan V, Halfon P, Fong A, Burnand B, Luthi JC, Saunders LD, Beck CA, Feasby TE, Ghali WA (2005). Coding algorithms for defining comorbidities in ICD-9-CM and ICD-10 administrative data. Med Care.

[28] Fulton BG, Ryerson CJ (2015). Managing comorbidities in idiopathic pulmonary fibrosis. Int J Gen Med.

[29] Cox DR (1972). Regression models and life-tables. J R Stat Soc Ser B Methodol.

[30] Divo M, Cote C, de Torres JP, Casanova C, Marin JM, Pinto-Plata V, Zulueta J, Cabrera C, Zagaceta J, Hunninghake G, Celli B (2012). Comorbidities and risk of mortality in patients with chronic obstructive pulmonary disease. Am J Respir Crit Care Med.

[31] Tibshirani R (1996). Regression shrinkage and selection via the lasso. J R Stat Soc.

[32] Tibshirani R (1997). The lasso method for variable selection in the cox model. Stat Med.

[33] Oyeyemi G, Oluwole Ogunjobi E, Idowu Folorunsho A (2015). On performance of shrinkage methods – a Monte Carlo study. Int J Stat Appl.

[34] Lockhart R, Taylor J, Tibshirani RJ, Tibshirani R (2014). A significance test for the lasso. Ann Stat.

[35] Piccirillo JF, Vlahiotis A, Barrett LB, Flood KL, Spitznagel EL, Steyerberg EW (2008). The changing prevalence of comorbidity across the age spectrum. Crit Rev Oncol Hematol.

[36] Charlson ME, Pompei P, Ales KL, MacKenzie CR (1987). A new method of classifying prognostic comorbidity in longitudinal studies: development and validation. J Chronic Dis.

[37] Kurth B-M (2012). Erste Ergebnisse aus der - Studie zur Gesundheit Erwachsener in Deutschland? (DEGS). Bundesgesundheitsbl Gesundheitsforsch Gesundheitsschutz.

[38] King CS, Nathan SD (2017). Idiopathic pulmonary fibrosis: effects and optimal management of comorbidities. Lancet Respir Med.

[39] Galie N, Humbert M, Vachiery JL, Gibbs S, Lang I, Torbicki A, Simonneau G, Peacock A, Vonk Noordegraaf A, Beghetti M (2015). ESC/ERS guidelines for the diagnosis and treatment of pulmonary hypertension: the joint task force for the diagnosis and treatment of pulmonary hypertension of the European Society of Cardiology (ESC) and the European Respiratory Society (ERS): endorsed by: Association for European Paediatric and Congenital Cardiology (AEPC), International Society for Heart and Lung Transplantation (ISHLT). Eur Heart J.

[40] Mermigkis C, Bouloukaki I, Antoniou K, Papadogiannis G, Giannarakis I, Varouchakis G, Siafakas N, Schiza SE (2015). Obstructive sleep apnea should be treated in patients with idiopathic pulmonary fibrosis. Sleep Breath.

[41] Lee JS, Ryu JH, Elicker BM, Lydell CP, Jones KD, Wolters PJ, King TE, Collard HR (2011). Gastroesophageal reflux therapy is associated with longer survival in patients with idiopathic pulmonary fibrosis. Am J Respir Crit Care Med.

[42] Johannson KA, Strambu I, Ravaglia C, Grutters JC, Valenzuela C, Mogulkoc N, Luppi F, Richeldi L, Wells AU, Vancheri C, Kreuter M (2017). Antacid therapy in idiopathic pulmonary fibrosis: more questions than answers?. Lancet Respir Med.

[43] Tomassetti S, Ruy JH, Gurioli C, Ravaglia C, Buccioli M, Tantalocco P, Decker PA, Cavazza A, Dubini A, Agnoletti V (2013). The effect of anticoagulant therapy for idiopathic pulmonary fibrosis in real life practice. Sarcoidosis Vasc Diffuse Lung Dis.

[44] Noth I, Anstrom KJ, Calvert SB, de Andrade J, Flaherty KR, Glazer C, Kaner RJ, Olman MA (2012). A placebo-controlled randomized trial of warfarin in idiopathic pulmonary fibrosis. Am J Respir Crit Care Med.

[45] Vedel-Krogh S, Nielsen SF, Nordestgaard BG (2015). Statin use is associated with reduced mortality in patients with interstitial lung disease. PLoS One.

[46] Schubert I, Köster I, Küpper-Nybelen J, Ihle P (2008). Versorgungsforschung mit GKV-Routinedaten. Bundesgesundheitsbl Gesundheitsforsch Gesundheitsschutz.

[47] O'Malley KJ, Cook KF, Price MD, Wildes KR, Hurdle JF, Ashton CM (2005). Measuring diagnoses: ICD code accuracy. Health Serv Res.

[48] du Bois RM, Weycker D, Albera C, Bradford WZ, Costabel U, Kartashov A, King TE, Lancaster L, Noble PW, Sahn SA (2011). Forced vital capacity in patients with idiopathic pulmonary fibrosis: test properties and minimal clinically important difference. Am J Respir Crit Care Med.

[49] Goh NS, Hoyles RK, Denton CP, Hansell DM, Renzoni EA, Maher TM, Nicholson AG, Wells AU (2017). Short-term pulmonary function trends are predictive of mortality in interstitial lung disease associated with systemic sclerosis. Arthritis Rheumatol.

[50] Girard N, Marchand-Adam S, Naccache JM, Borie R, Urban T, Jouneau S, Marchand E, Ravel AC, Kiakouama L, Etienne-Mastroianni B (2014). Lung cancer in combined pulmonary fibrosis and emphysema: a series of 47 western patients. J Thorac Oncol.

