# Supplementary figures and images for: Associations between comorbidities, their treatment and survival in patients with interstitial lung diseases – a claims data analysis
Source: Respir Res. 2018 Apr 25;19:73. doi: 10.1186/s12931-018-0769-0 (PMC5918773; doi:10.1186/s12931-018-0769-0)

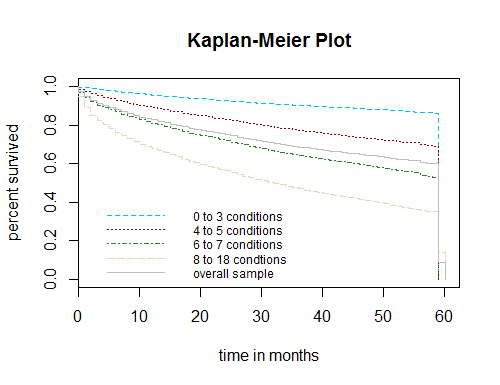


Figure S1: Kaplan–Meier plots according to comorbidity quartile

Supplement: Supplementary file 1 — Figure S1. Kaplan–Meier plots according to comorbidity quartile. (DOC 35 kb) [file 12931_2018_769_MOESM1_ESM.doc]
