# Supplementary material for: Associations between comorbidities, their treatment and survival in patients with interstitial lung diseases – a claims data analysis
Source: Respir Res. 2018 Apr 25;19:73. doi: 10.1186/s12931-018-0769-0 (PMC5918773; doi:10.1186/s12931-018-0769-0)
Supplement: Supplementary file 2 — Table S1. Baseline characteristics for the population stratified by ILD subtype. (DOC 81 kb) [file 12931_2018_769_MOESM2_ESM.doc]

Table S1: Baseline characteristics for the population stratified by ILD subtype

|  | **SARC** | **IIP** | **OFI** | **DAI** | **PNE** | **RAP** | **EPP** | **HP** | **CTD** |
| --- | --- | --- | --- | --- | --- | --- | --- | --- | --- |
| % | 24.7 | 39.3 | 19.5 | 1.1 | 4.3 | 1.3 | 4.1 | 2.6 | 3.1 |
| Ø age at confirmed diagnosis | 55.4 | 71.6 | 67.6 | 69 | 72.7 | 68.4 | 64.8 | 62.9 | 62.2 |
| % Male gender | 47.1 | 60.1 | 55.9 | 53.1 | 93.7 | 60.6 | 59.9 | 44.2 | 34.3 |
| Ø time under observation in months | 34.8 | 26.2 | 29.5 | 26.7 | 28.7 | 20.6 | 30.6 | 34.7 | 31.3 |
| % Dead at end of observation period | 9.5 | 68.4 | 32.5 | 41.5 | 35.6 | 67.9 | 30.3 | 16.0 | 28.9 |
| Ø Number of comorbidities | 3.5 | 5.6 | 5.1 | 6.3 | 5.4 | 6.5 | 4.7 | 4.3 | 6.1 |
| % Congestive heart failure | 14.2 | 31.0 | 29.8 | 38.1 | 32.8 | 30.8 | 23.1 | 22.4 | 29.0 |
| % Cardiac arrhythmia | 15.6 | 32.1 | 27.3 | 46.7 | 30.6 | 28.9 | 23.5 | 23.3 | 25.1 |
| % Valvular disease | 8.5 | 18.3 | 14.5 | 18.7 | 18.9 | 12.9 | 10.7 | 12.5 | 19.7 |
| % Pulmonary circulation disorders | 5.3 | 17.0 | 11.8 | 14.3 | 12.9 | 13.8 | 7.6 | 10.2 | 18.9 |
| % Peripheral vascular disorders | 9.1 | 22.7 | 19.0 | 25.3 | 23.2 | 31.5 | 20.2 | 10.3 | 17.3 |
| % Hypertension uncomplicated | 42.4 | 55.5 | 53.8 | 57.7 | 58.7 | 56.3 | 52.0 | 54.8 | 50.9 |
| % Hypertension complicated | 10.4 | 19.5 | 16.0 | 20.6 | 17.8 | 16.0 | 12.5 | 11.9 | 18.3 |
| % Chronic pulmonary disease | 40.3 | 62.9 | 57.2 | 43.5 | 74.8 | 59.1 | 62.8 | 68.8 | 41.3 |
| % Diabetes uncomplicated | 14.1 | 19.6 | 17.7 | 20.9 | 18.4 | 19.4 | 15.0 | 16.3 | 14.5 |
| % Diabetes complicated | 10.4 | 16.4 | 13.6 | 13.8 | 15.5 | 11.1 | 11.4 | 11.1 | 11.4 |
| % Hypothyroidism | 12.5 | 11.8 | 12.7 | 18.2 | 8.3 | 11.5 | 13.2 | 18.9 | 18.9 |
| % Renal failure | 11.2 | 23.6 | 17.9 | 31.9 | 21.8 | 15.0 | 14.0 | 35.4 | 35.4 |
| % Liver disease | 13.6 | 14.4 | 14.0 | 14.0 | 14.5 | 14.0 | 12.2 | 14.2 | 14.2 |
| % Metastatic cancer | 3.2 | 5.5 | 8.5 | 17.0 | 6.7 | 46.6 | 8.8 | 2.1 | 3.4 |
| % Solid tumour (without metastasis) | 13.1 | 13.1 | 16.0 | 21.9 | 15.0 | 34.9 | 14.4 | 8.2 | 7.5 |
| % Rheumatoid arthritis/ Con Connective tissue disorder | 9.1 | 13.7 | 12.0 | 24.6 | 7.9 | 6.3 | 8.0 | 10.6 | 96.3 |
| % Coagulopathy | 4.0 | 8.0 | 7.7 | 13.8 | 7.2 | 6.0 | 7.3 | 3.4 | 10.4 |
| % Obesity | 21.3 | 17.2 | 19.4 | 14.9 | 13.8 | 16.1 | 22.8 | 15.0 | 15.0 |
| % Weight loss | 3.1 | 6.4 | 5.5 | 5.2 | 6.3 | 10.1 | 7.0 | 3.5 | 6.8 |
| % Fluid and electrolyte disorders | 8.2 | 21.1 | 16.5 | 18.8 | 17.5 | 21.8 | 14.2 | 10.2 | 26.1 |
| % Depression | 19.5 | 19.8 | 21.1 | 19.9 | 15.3 | 23.3 | 20.5 | 22.7 | 26.4 |
| % IHD | 16.7 | 40.0 | 32.4 | 41.0 | 42.8 | 39.2 | 30.2 | 24.5 | 25.0 |
| % GERD | 10.0 | 15.8 | 16.1 | 15.5 | 14.5 | 18.1 | 13.0 | 16.4 | 20.4 |
| % OSAS | 6.1 | 6.3 | 7.2 | 7.9 | 6.5 | 4.3 | 6.1 | 7.5 | 25.0 |

SARC = sarcoidosis (n=9 106), IIP = idiopathic interstitial pneumonia (n=14 453), OFI = other fibrosing ILDs (n=7 187), DAI = drug-associated ILD (n=407), PNE = pneumoconiosis (n=1 579), RAP = radiation-associated pneumonitis (n=464), EEP = eosinophilic pneumonia (n=1 518), HP = hypersensitivity pneumonitis (n=967), CTD = connective tissue-associated ILD (n=1 140)
