# Supplementary material for: Associations between comorbidities, their treatment and survival in patients with interstitial lung diseases – a claims data analysis
Source: Respir Res. 2018 Apr 25;19:73. doi: 10.1186/s12931-018-0769-0 (PMC5918773; doi:10.1186/s12931-018-0769-0)
Supplement: Supplementary file 3 — Table S2. Prescription patterns of comorbidity-relevant medication at the subtype level. (DOC 75 kb) [file 12931_2018_769_MOESM3_ESM.doc]

Table S2: Prescription patterns of comorbidity-relevant medication at the subtype level

|  |  | **SARC** | **IIP** | **OFI** | **DAI** | **PNE** | **RAP** | **EEP** | **HP** | **CTD** |
| --- | --- | --- | --- | --- | --- | --- | --- | --- | --- | --- |
| *Treatment of heart insufficiency/cardiac arrhythmia* | | 17.1 | 38.2 | 32.5 | 47.4 | 31.1 | 31.9 | 25.7 | 27.2 | 33.0 |
| Digitalis glycosides | | 1.9 | 5.1 | 4.5 | 6.9 | 6.1 | 6.7 | 3.6 | 4.1 | 3.2 |
| Anti-arrhythmic drugs | | 0.4 | 1.8 | 1.8 | 12.5 | 1.1 | 0.9 | 1.3 | 1.0 | 1.1 |
| Diuretic drugs | | 16.3 | 36.2 | 30.5 | 41.0 | 28.8 | 28.7 | 24.2 | 25.0 | 31.8 |
| *Treatment of IHD/hypertension* | | 40.5 | 58.3 | 55.3 | 61.2 | 59.1 | 51.9 | 49.5 | 53.3 | 52.0 |
| Statins | | 10.7 | 18.9 | 17.3 | 16.7 | 18.6 | 15.3 | 15.5 | 13.7 | 22.8 |
| Beta-blockers | | 22.1 | 33.7 | 32.5 | 39.1 | 32.3 | 31.0 | 26.9 | 29.0 | 11.0 |
| ACE inhibitors | | 18.0 | 28.7 | 25.8 | 27.5 | 34.6 | 24.8 | 25.1 | 23.2 | 8.5 |
| Antiotensin-I-antagonists | | 11.0 | 13.1 | 13.8 | 17.2 | 12.1 | 9.3 | 12.7 | 15.3 | 7.2 |
| *Treatment with anti-clotting medication* | | 12.4 | 24.6 | 22.6 | 32.4 | 27.2 | 28.2 | 18.7 | 15.1 | 62.8 |
| Antiplatelet drugs | | 6.3 | 13.6 | 11.3 | 12.5 | 15.5 | 14.4 | 10.0 | 8.4 | 61.2 |
| Vitamin-K antagonists | | 4.3 | 7.1 | 7.8 | 13.5 | 7.8 | 11.9 | 5.7 | 3.4 | 1.1 |
| Heparin(-derivates) | | 3.5 | 7.3 | 6.7 | 11.1 | 8.4 | 5.6 | 6.5 | 5.8 | 13.4 |
| *Treatment with anti-acid drugs* | | 34.3 | 40.3 | 41.8 | 52.8 | 34.5 | 51.3 | 35.6 | 40.2 | 11.6 |
| Proton pump inhibitors | | 33.5 | 20.1 | 78.5 | 52.3 | 33.5 | 48.5 | 34.4 | 39.6 | 18.8 |
| H2-blockers | | 0.9 | 1.3 | 1.5 | 1.0 | 1.3 | 2.6 | 1.4 | 1.1 | 4.6 |
| *Treatment with anti-depressants* | | 6.2 | 22.2 | 193.4 | 2.9 | 31.9 | 4.9 | 14.7 | 9.0 | 7.3 |
| *Treatment with anti-diabetic drugs* | | 14.1 | 17.4 | 16.8 | 16.2 | 15.2 | 19.6 | 14.0 | 14.0 | 4.0 |
| *Treatment of COPD* | | 22.8 | 33.0 | 30.4 | 21.9 | 35.3 | 37.7 | 38.3 | 39.5 | 9.0 |
| Long-acting beta2 agonists (LABA) | | 4.0 | 8.4 | 7.4 | 3.7 | 10.4 | 10.1 | 10.7 | 9.1 | 0.1 |
| Long-acting muscarinic antagonists (LAMA) | | 5.1 | 16.2 | 13.4 | 10.3 | 17.8 | 22.6 | 19.2 | 13.3 | 6.3 |
| Inhaled corticosteroids (ICS) | | 8.1 | 7.3 | 7.5 | 4.9 | 5.6 | 9.1 | 9.7 | 12.3 | 4.0 |
| Combination product LABA/ICS | | 11.2 | 15.2 | 13.9 | 10.6 | 15.6 | 15.1 | 17.0 | 20.2 | 9.0 |
| Combination product LABA/LAMA | | 0.1 | 0.1 | 0.1 | 0.0 | 0.3 | 0.0 | 0.0 | 0.0 | 0.1 |
| *Treatment with specific PH drugs* | | 0.2 | 0.8 | 0.7 | 0.2 | 0.2 | 0.2 | 0.3 | 0.4 | 6.3 |

All figures reflect percentages

SARC = sarcoidosis (n=9 106), IIP = idiopathic interstitial pneumonia (n=14 453), OFI = other fibrosing ILDs (n=7 187), DAI = drug-associated ILD (n=407), PNE = pneumoconiosis (n=1 579), RAP = radiation-associated pneumonitis (n=464), EEP = eosinophilic pneumonia (n=1 518), HP = hypersensitivity pneumonitis (n=967), CTD = connective tissue-associated ILD (n=1 140)
