# Supplementary material for: Associations between comorbidities, their treatment and survival in patients with interstitial lung diseases – a claims data analysis
Source: Respir Res. 2018 Apr 25;19:73. doi: 10.1186/s12931-018-0769-0 (PMC5918773; doi:10.1186/s12931-018-0769-0)
Supplement: Supplementary file 4 — Table S3. ILD subtype-specific hazard ratios as per the comorbidity-only Cox model. (DOC 97 kb) [file 12931_2018_769_MOESM4_ESM.doc]

Table S3: ILD subtype-specific hazard ratios as per the comorbidity-only Cox model

| **Variable** | **SARC** | **IIP** | **OFI** | **DAI** | **PNE** | **RAP** | | **EPP** | | **HP** | | **CTD** | |
| --- | --- | --- | --- | --- | --- | --- | --- | --- | --- | --- | --- | --- | --- |
| Female gender | 0.66 | 0.71 | 0.64 | 0.55 | 0.93 | 0.68 | | 0.79 | | 0.51 | | 0.58 | |
| Ø age at diagnosis | 1.06 | 1.04 | 1.04 | 1.03 | 1.05 | 1.01 | | 1.04 | | 1.05 | | 1.04 | |
| Congestive heart failure treated | 1.77 | 1.48 | 1.41 | -/- | 1.37 | 1.22^ns^ | | 1.49 | | 1.32^ns^ | | 1.85 | |
| Congestive heart failure untreated | 2.97 | 1.59 | 1.42 | 0.62^ns^ | 2.01 | 1.50^ns^ | | 2.13 | | 1.20^ns^ | | 1.90 | |
| Cardiac arrhythmia treated | 1.09^ns^ | 0.97^ns^ | 0.95^ns^ | 0.59^ns^ | 1.29 | 1.22 | | 1.11^nss^ | | 0.68^ns^ | | 1.11^ns^ | |
| Cardiac arrhythmia untreated | 1.08^ns^ | 1.11 | 1.15 | 1.25 | 1.38 | 2.17 | | 1.19^nss^ | | -/- | | 1.25^ns^ | |
| Valvular disease treated | -/- | 0.94^ns^ | 0.89^ns^ | -/- | 1.01^ns^ | 1.22 | | -/- | | -/- | | 0.57 | |
| Valvular disease untreated | -/- | 1.06^ns^ | 1.04^ns^ | -/- | 1.31^ns^ | 1.71^ns^ | | 1.15^ns^ | | -/- | | 1.17^ns^ | |
| Pulmonary hypertension treated | 2.01 | 1.35 | 1.33 | 0.74^ns^ | -/- | -/- | | 1.22^ns^ | | 3.01 | | 2.25 | |
| Pulmonary hypertension untreated | 1.53 | 1.41 | 1.41 | 1.28^ns^ | 1.03n.s | -/- | | 1.00^ns^ | | 2.00^ns^ | | 1.75 | |
| Peripheral vascular disorders treated | -/- | 1.07^ns^ | 1.07^ns^ | 1.26^ns^ | -/- | 1.52 | | 1.56 | | 1.39^ns^ | | -/- | |
| Peripheral vascular disorders untreated | 1.09^ns^ | 1.08^ns^ | 1.06^ns^ | -/- | 1.02^n.s^ | 1.47 | | 1.27^ns^ | | 2.72 | | -/- | |
| Hypertension without complications treated | 1.20 | 1.04^ns^ | 0.90 | -/- | -/- | -/- | | -/- | | -/- | | -/-. | |
| Hypertension without complications untreated | 1.46 | 1.20 | 1.50 | -/- | 1.02^ns^ | 0.79^ns^ | | -/- | | 1.01^ns^ | | 1.04^ns^ | |
| Hypertension with complications treated | -/- | 0.95^ns^ | 0.91^ns^ | 0.61^ns^ | 1.65 | -/- | | 0.73 | | -/- | | 1.44 | |
| Hypertension with complications untreated | 0.86^ns^ | -/ | -/- | -/- | -/- | | -/- | | 0.44^ns^ | | -/-. | |  |
| COPD treated | -/- | 0.86 | 0.86 | -/- | -/- | -/- | | -/- | | 0.84^ns^ | | 1.04^ns^ | |
| COPD untreated | -/- | 0.95^ns^ | 0.92^ns^ | -/- | 0.89^ns^ | -/- | | 0.99^ns^ | | 1.23^ns^ | | -/- | |
| Diabetes without complications treated | 1.01^ns^ | 1.10^ns^ | 0.97^ns^ | -/- | 0.88 | -/- | | -/- | | -/- | | 0.97^ns^ | |
| Diabetes without complications untreated | -/- | 1.19 | 1.17 | -/- | -/- | -/- | | 1.06^ns^ | | -/- | | -/- | |
| Diabetes with complications treated | 1.26 | 0.87 | 1.12^ns^ | -/- | 1.35 | 1.45 | | -/- | | 0.74^ns^ | | 1.11^ns^. | |
| Diabetes with complications untreated | 1.08^ns^ | 1.26 | 1.25 | 0.90^ns^ | 1.37^ns^ | 0.61^n.s^ | | 1.02^ns^ | | -/- | | -/- | |
| Hypothyroidism | -/- | 1.06 | 0.91^ns^ | 0.83^ns^ | 1.39 | -/- | | -/- | | 0.68^ns^ | | -/- | |
| Renal failure | 1.39 | 2.29 | 1.35 | 1.05^ns^ | 1.26 | -/- | | 1.48 | | -/- | | 1.16^ns^ | |
| Liver disease | -/- | 1.12 | -/- | -/- | -/- | -/- | | 1.15^ns^ | | -/- | | 1.18^ns^ | |
| Metastatic carcinoma | 3.22 | 0.89 | 2.99 | 2.74 | 2.45 | 2.01 | | 2.57 | | 6.49 | | 1.50^ns^ | |
| Solid tumour without metastasis | 1.35 | 1.36 | 1.12 | 1.41^ns^ | -/- | -/- | | 1.23^ns^ | | 1.05^ns^ | | 1.29^ns^ | |
| Rheumatoid arthritis | 0.83^ns^ | 0.89 | 0.82 | -/- | -/- | -/- | | -/- | | -/- | | -/- | |
| Coagulopathy | 1.62 | 1.36 | 1.39 | 1.46^ns^ | 1.16^ns^ | -/- | | 1.38 | | 1.52^ns^ | | 2.10 | |
| Obesity | -/- | 0.85 | 0.85 | -/- | -/- | 0.70^ns^ | | -/- | | -/- | | 0.97^ns^ | |
| Weight loss | 1.50 | 1.55 | 1.61 | 1.75 | 2.02 | 1.47^ns^ | | 1.81 | | 2.15 | | -/- | |
| Fluid and electrolyte disorders | 2.19 | 1.71 | 1.58 | -/- | 1.92^ns^ | 1.38^ns^ | | 2.11 | | 1.41^nss^. | | 1.7 | |
| Deficiency anaemia | 1.50 | 1.04 | 1.18 | -/- | -/- | -/- | | 1.23^ns^ | | 2.10 | | -/- | |
| Depression treated | -/- | -/- | 1.19 | -/- | -/- | -/- | | 0.97^ns^ | | -/- | | -/- | |
| Depression untreated | -/- | 0.97^ns^ | 0.90^ns^ | -/- | 0.86^ns^ | -/- | | -/- | | -/- | | 0.93^ns^ | |
| IHD treated | -/- | 0.95^ns^ | 1.06^ns^ | -/- | -/- | -/- | | 0.71 | | 0.58^ns^ | | 1.28^ns^ | |
| IHD untreated | 1.20^ns^s. | 1.31 | 1.35 | 0.99^ns^ | 1.07^n.s^ | -/- | | 1.38 | | -/- | | 2.12 | |
| GERD treated | -/- | 1.00^ns^ | 0.96^ns^ | -/- | -/- | -/- | | 0.95^ns^ | | -/- | | 0.85^ns^ | |
| GERD untreated | -/- | -/- | 0.76 | -/- | 1.21^ns^ | 1.09^ns^ | | -/- | | -/- | | -/- | |
| OSAS | 0.60 | 0.69 | 0.79 | -/- | 0.62 | -/- | | 1.22^ns^ | | 0.58^ns^ | | 0.80^ns^ | |
| Lung cancer | 1.86 | 1.77 | 1.86 | 1.89 | 1.54 | 1.75 | | 2.67 | | -/- | | 2.06 | |

SARC = sarcoidosis (n=9 106), IIP = idiopathic interstitial pneumonia (n=14 453); OFI = other fibrosing ILDs (n=7 187), DAI = drug-associated ILD (n=407), PNE = pneumoconiosis (n=1 579), RAP = radiation-associated pneumonitis (n=464); EEP = eosinophilic pneumonia (n=1 518); HP = hypersensitivity pneumonitis (n=967), CTD = connective tissue-associated ILD (n=1 140)

not significant ^‘ns’^.
