# Supplementary material for: Associations between comorbidities, their treatment and survival in patients with interstitial lung diseases – a claims data analysis
Source: Respir Res. 2018 Apr 25;19:73. doi: 10.1186/s12931-018-0769-0 (PMC5918773; doi:10.1186/s12931-018-0769-0)
Supplement: Supplementary file 5 — Figure S3. Pneumoconios-, Drug-associated ILD-, Radiation-asscociated pneumonitis-, Eosinophilic pneumonia-, HP- and CTD-specific comorbidomes based on results of the LASSO selection for the comorbidity-only Cox model. (DOC 648 kb) [file 12931_2018_769_MOESM5_ESM.doc]

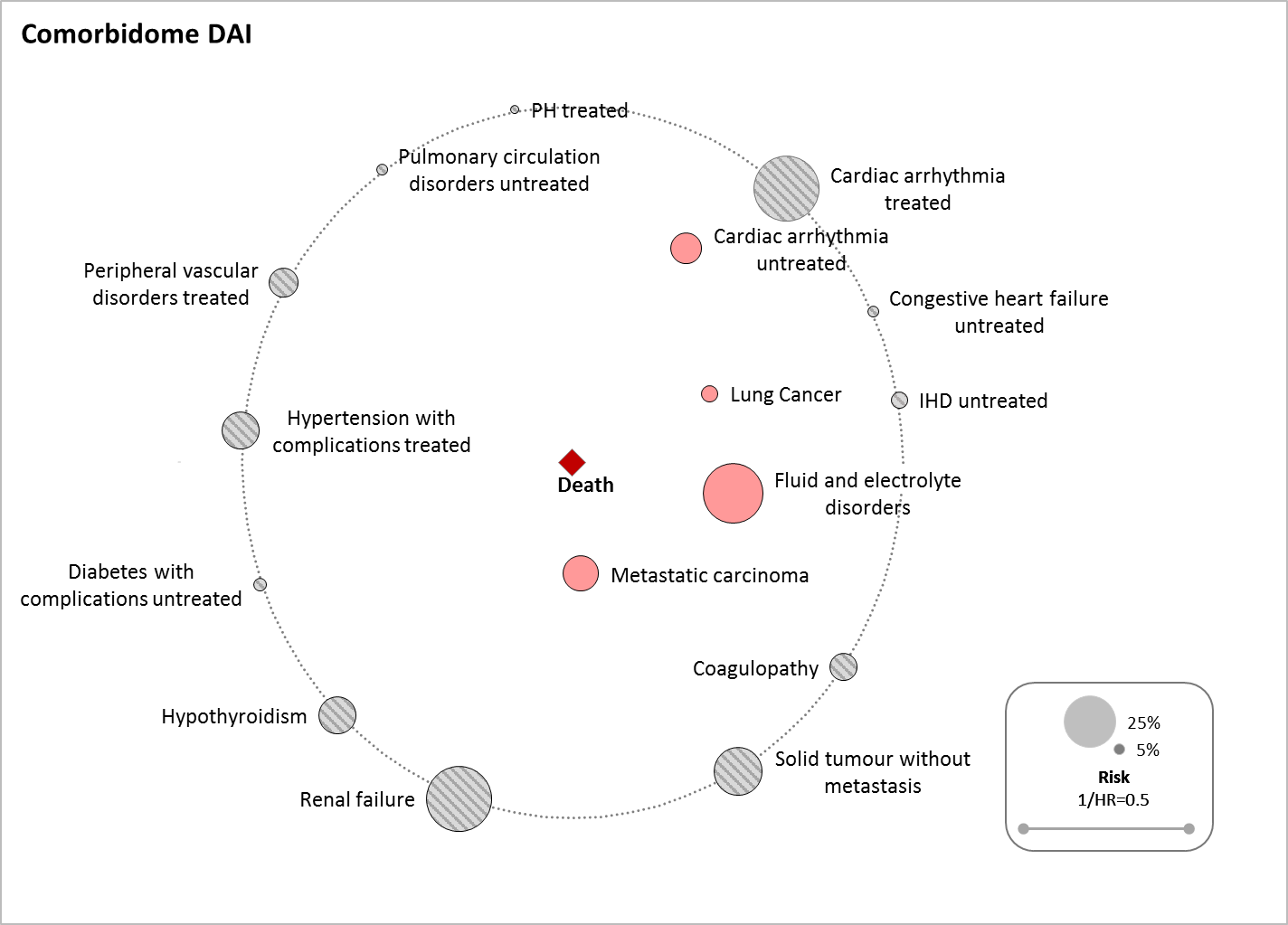

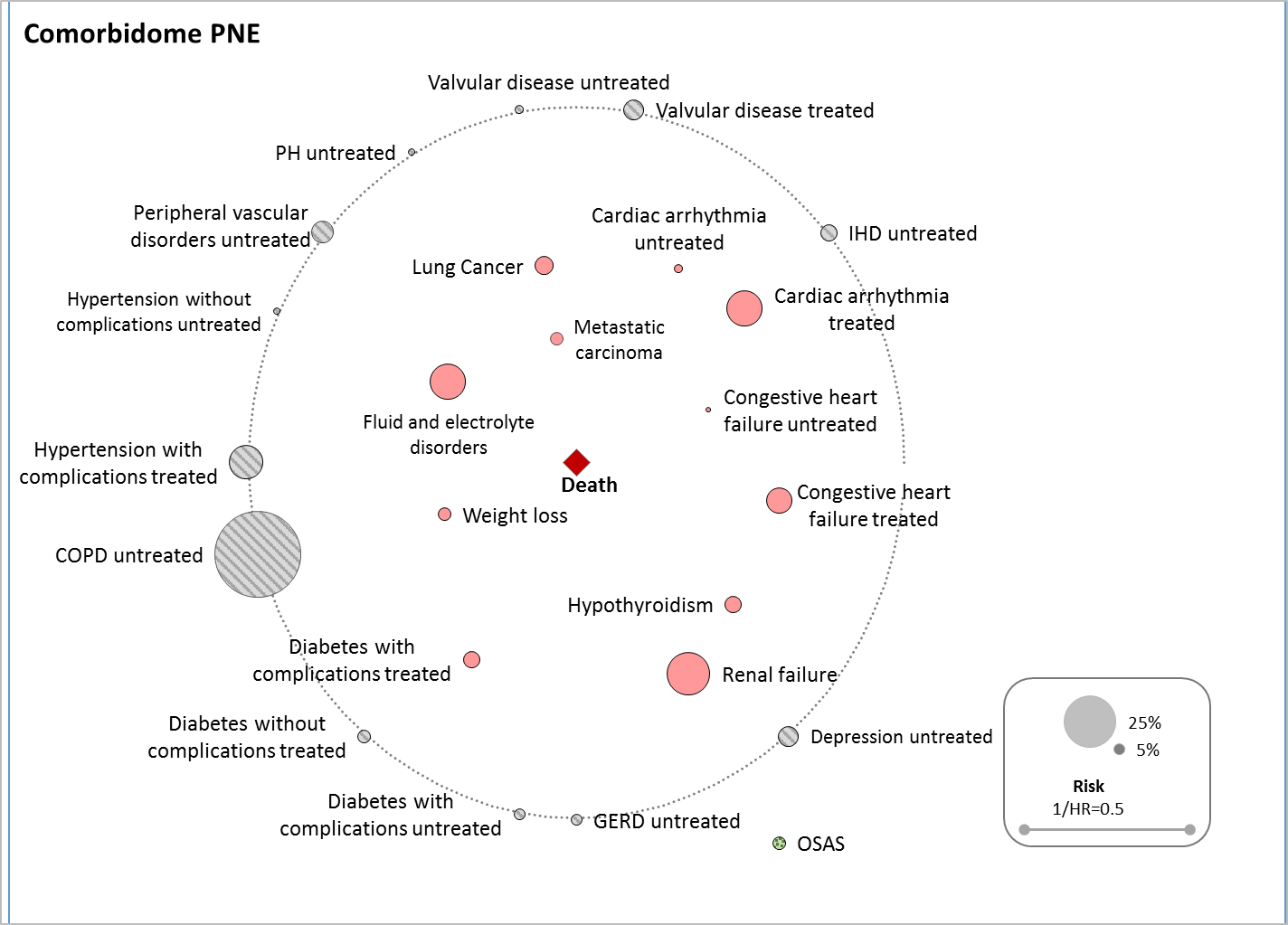

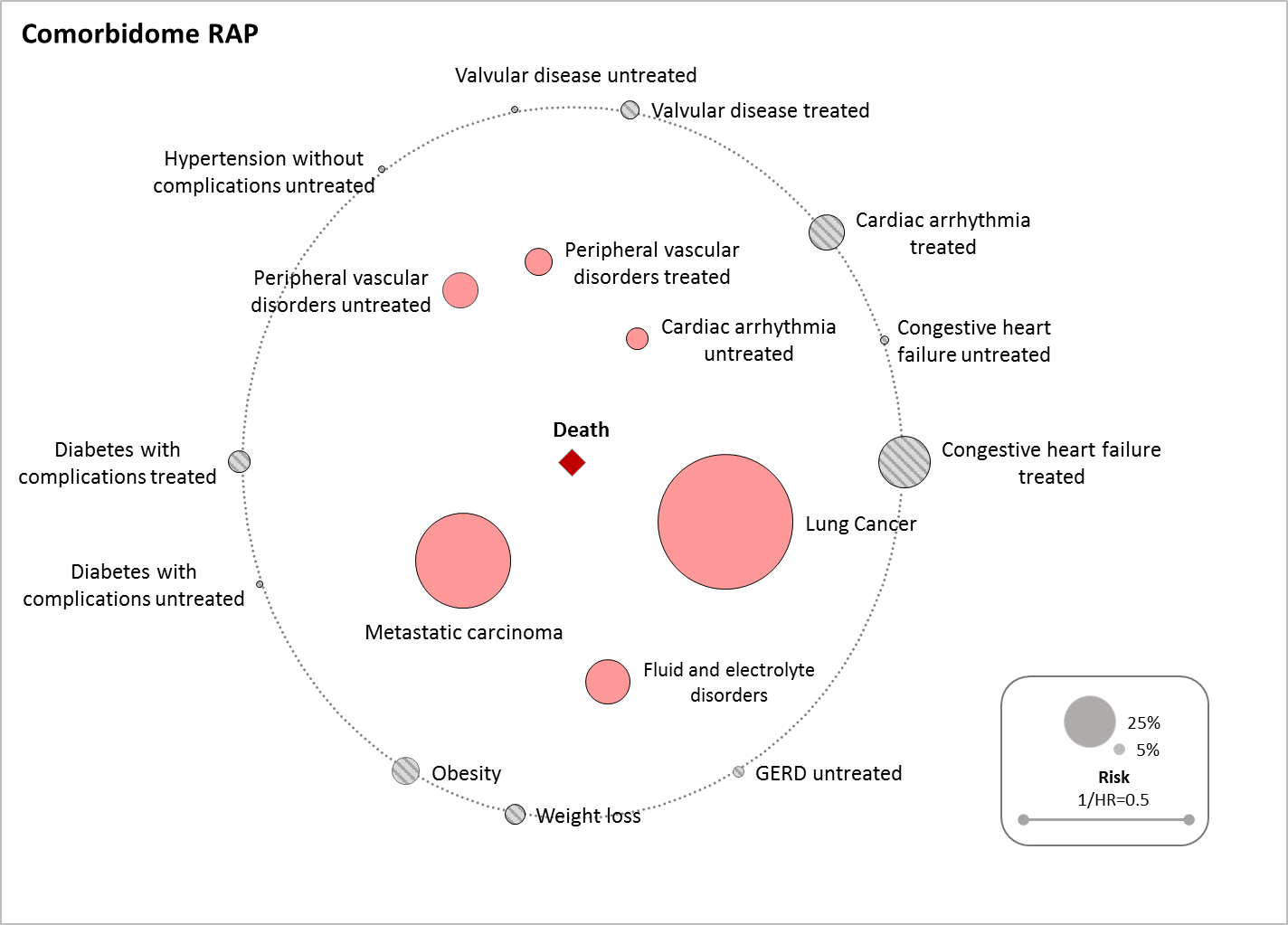

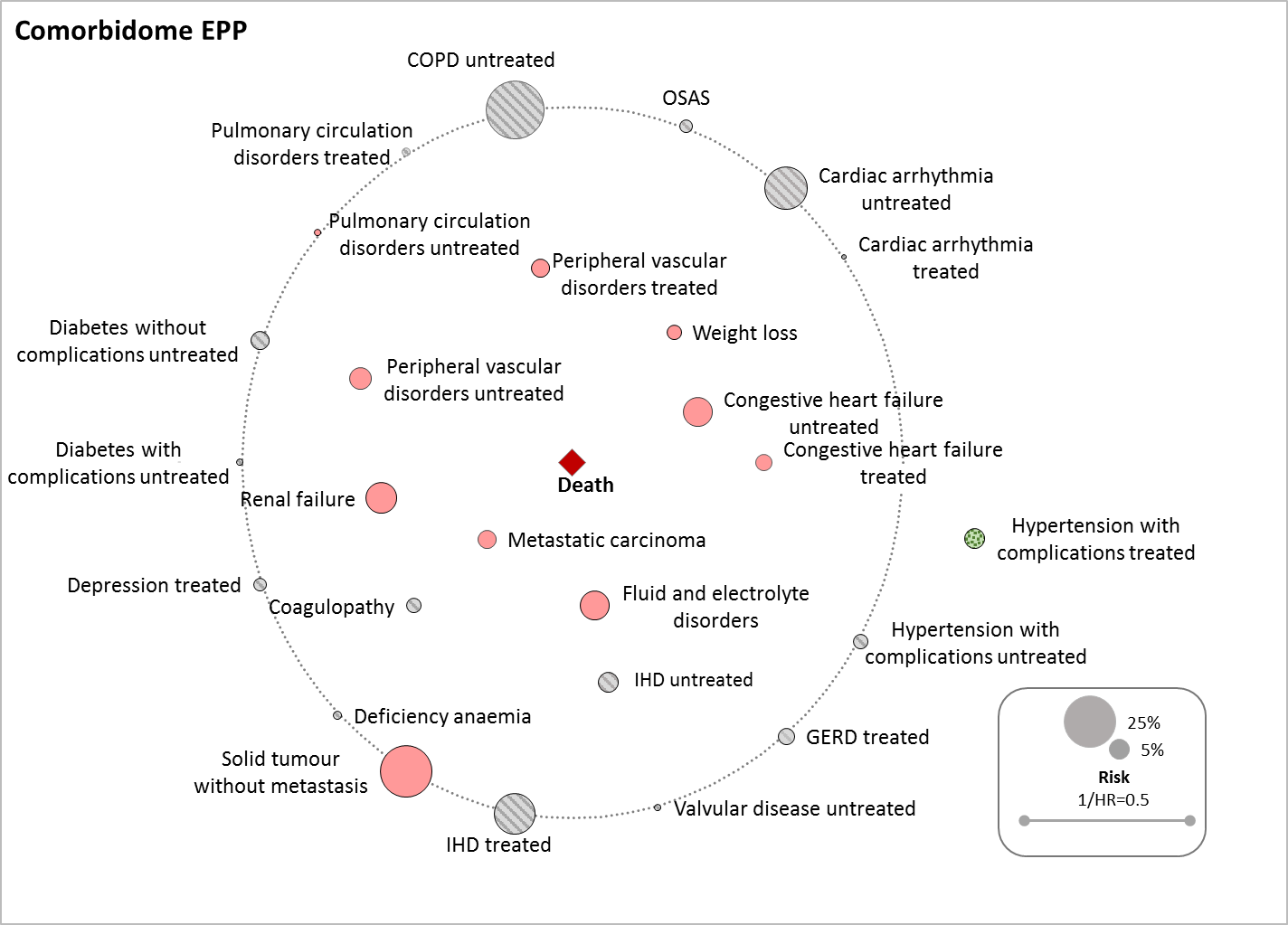

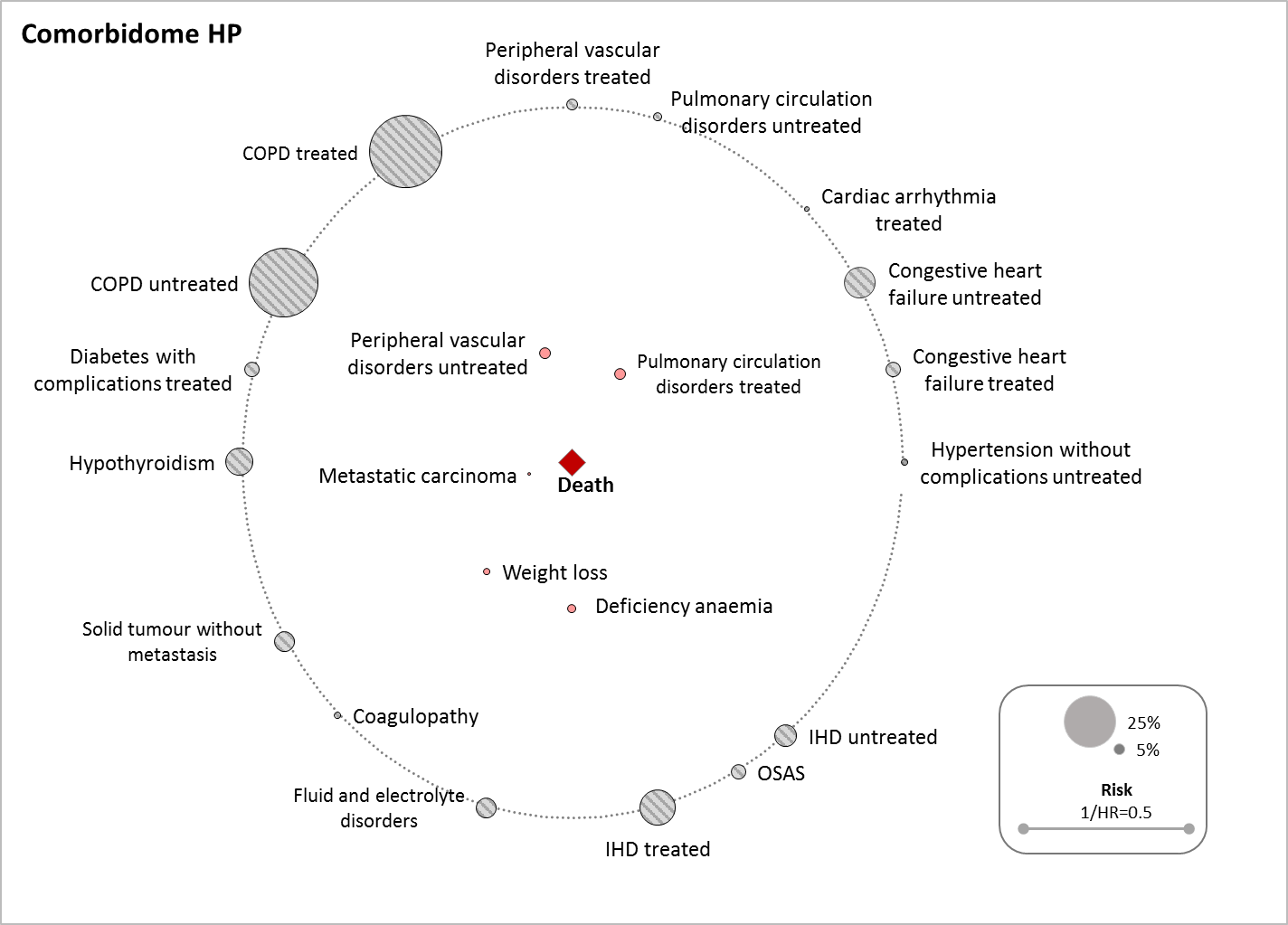

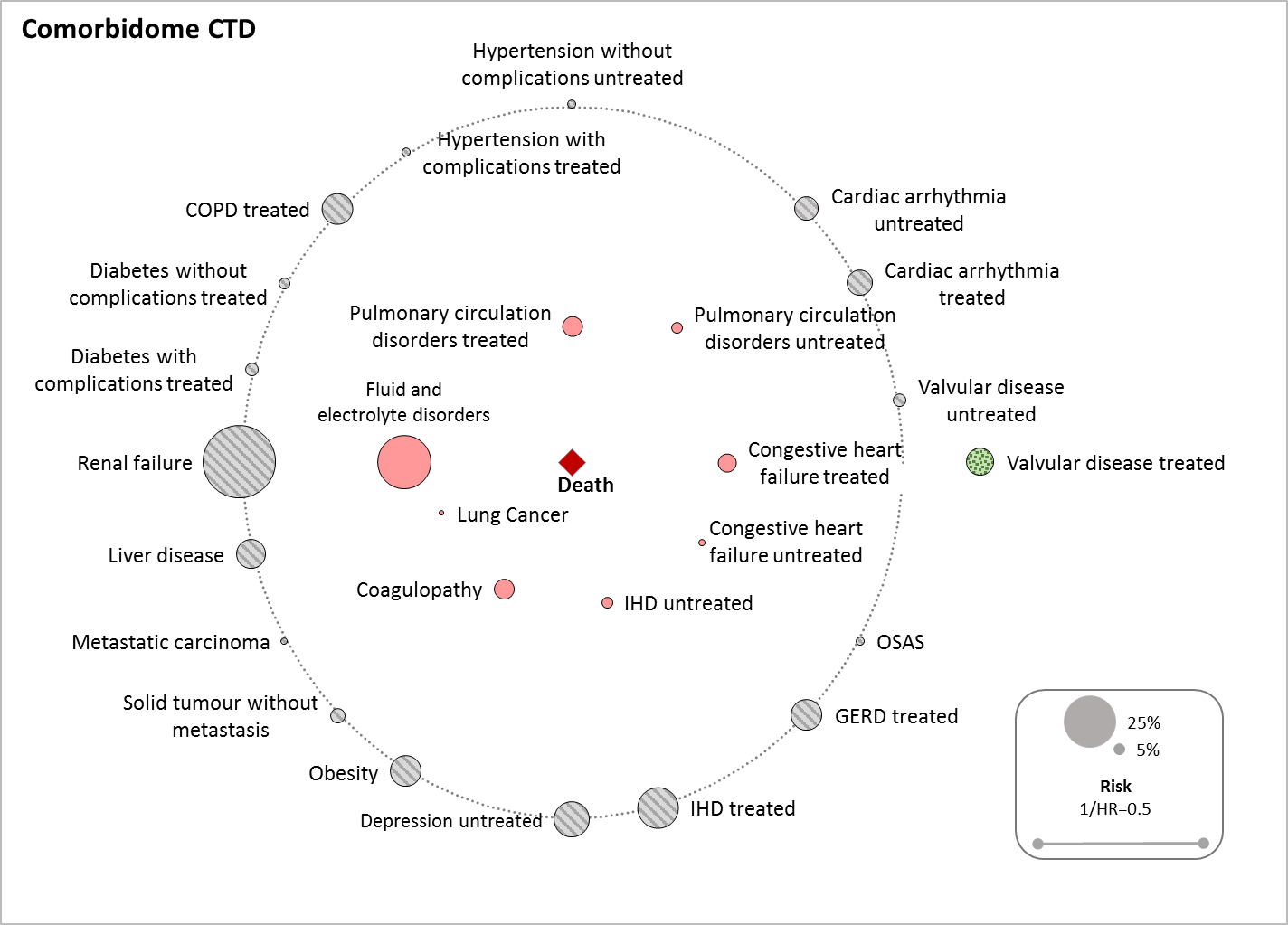


Graphic expression of comorbid conditions with prevalence ≥ 5% that were selected in the LASSO procedure for the comorbidity only model. The area of the circles relates to the prevalence of the condition. Conditions with a significant positive impact (hazard ratio (HR) ≤ 1) are fully outside the dotted orbit. Those with a significant negative impact on survival (HR ≥1) are fully inside the dotted orbit with proximity to the centre (death) reflecting the strength of the association (1/HR). Conditions located on the dotted line did not have a statistically significant survival impact. DAI = drug-associated ILD (n=407), PNE = pneumoconiosis (n=1 579), RAP = radiation-associated pneumonitis (n=464), EEP = eosinophilic pneumonia (n=1 518), HP = hypersensitivity pneumonitis (n=967), CTD = connective tissue-associated ILD (n=1 140)

Figure S3: Pneumoconios-, Drug-associated ILD-, Radiation-associated pneumonitis-, Eosinophilic pneumonia-, HP- and CTD-specific comorbidomes based on results of the LASSO selection for the comorbidity-only Cox model
