# Supplementary material for: Associations between comorbidities, their treatment and survival in patients with interstitial lung diseases – a claims data analysis
Source: Respir Res. 2018 Apr 25;19:73. doi: 10.1186/s12931-018-0769-0 (PMC5918773; doi:10.1186/s12931-018-0769-0)
Supplement: Supplementary file 6 — Table S4. Baseline characteristics of the study sample within Sensitivity Analysis 2. (DOC 56 kb) [file 12931_2018_769_MOESM6_ESM.doc]

Table S4: Baseline characteristics of the study sample within Sensitivity Analysis 2

|  |  | **Study sample** | | | | |
| --- | --- | --- | --- | --- | --- | --- |
|  | | | **Main analysis** | | ***SA 2*** | |
| N | | | 36 821 | % | *21 581* |  |
|  | Idiopathic interstitial pneumonias (IIP) | | 14 453 | 39.3 | *8 627* | *40.0* |
|  | Other fibrosing ILDs (OFI) | | 7 187 | 19.5 | *3 142* | *14.6* |
|  | Sarcoidosis (SAR) | | 9 106 | 24.7 | *7 354* | *34.1* |
|  | Drug-associated ILDs (DAI) | | 407 | 1.1 | *137* | *0.6* |
|  | Pneumoconiosis (PNE) | | 1 579 | 4.3 | *1 011* | *4.7* |
|  | Radiation-associated pneumonitis (RAP) | | 464 | 1.3 | *164* | *0.8* |
|  | Eosinophilic pneumonia (EPP) | | 1 518 | 4.1 | *440* | *2.0* |
|  | Hypersensitivity pneumonitis (HP) | | 967 | 2.6 | *487* | *2.3* |
|  | Connective tissue disease-associated ILD (CTD) | | 1 140 | 3.1 | *219* | *1.0* |
| Ø age at diagnosis (SD) | | | 66.0 | 14.6 | *65.8* | *15.0* |
| Male gender | | | 20 704 | 56.2 | *12 123* | *56.2* |
| Ø time under observation in months (SD) | | | 29.6 | 0.31 | *27.3* | *0.3* |
| Dead at end of observation period | | | 11 422 | 31,0 | *7 295* | *33.8* |
| Median number of comorbidities (IQR)* | | | 5.0 | [3;7] | *4* | *[2;7]* |

All figures as N (%) unless reported otherwise
IQR = interquartile range

SD = Standard Deviation

SA = Sensitivity analysis
*31 comorbid conditions from the Elixhauser Index amended by lung cancer, PH, GERD, IHD, thrombosis and OSAS yields a maximum of 37
