# Supplementary material for: Associations between comorbidities, their treatment and survival in patients with interstitial lung diseases – a claims data analysis
Source: Respir Res. 2018 Apr 25;19:73. doi: 10.1186/s12931-018-0769-0 (PMC5918773; doi:10.1186/s12931-018-0769-0)
Supplement: Supplementary file 7 — Table S5. Prescription patterns of comorbidity-relevant medication according to sensitivity analyses. (DOC 74 kb) [file 12931_2018_769_MOESM7_ESM.doc]

Table S5: Prescription patterns of comorbidity-relevant medication according to sensitivity analyses

| **Individuals with prescriptions of therapeutic agents for comorbid conditions** | | | | | | | |
| --- | --- | --- | --- | --- | --- | --- | --- |
|  |  | **Main** | **%** | **SA 1** | **%** | **SA 2** | **%** |
| Treatment of heart insufficiency/cardiac arrhythmia | | 11 267 | 30.6 | 11 359 | 30.8 | 6 245 | 28.9 |
|  | Digitalis glycosides (ATC code C01AA) | 1 516 | 4.1 | 995 | 2.7 | 3 317 | 15.4 |
|  | Anti-arrhythmic drugs (ATC code C01BD) | 541 | 1.5 | 434 | 1.2 | 6 191 | 28.7 |
|  | Diuretic drugs (ATC code C03) | 10 631 | 28.9 | 10 317 | 28.0 | 5 073 | 23.5 |
| Treatment of cardiovascular disease | | 19 376 | 52.6 | 22 081 | 60.0 | 10 967 | 50.8 |
|  | Statins (ATC code C10AA) | 5 881 | 16.0 | 3 402 | 9.2 | 3 317 | 15.4 |
|  | Beta-blockers (ATC code C07) | 11 038 | 30.0 | 9 402 | 25.5 | 6 191 | 28.7 |
|  | ACE inhibitors (ATC codes C09AA, C09B) | 9 309 | 25.3 | 8 704 | 23.6 | 5 073 | 23.5 |
|  | Antiotensin-I-antagonists (ATC codes C09CA, C09D) | 4 664 | 12.7 | 3 562 | 9.7 | 2 701 | 12.5 |
| Treatment with anti-clotting medication | | 7 692 | 20.9 | 8 571 | 23.3 | 4 122 | 19.1 |
|  | Antiplatetlet drugs (ATC code B01AC) | 4 071 | 11.1 | 4 769 | 13.0 | 2 239 | 10.4 |
|  | Vitamin-K antagonists (ATC code B01AA) | 2 428 | 6.6 | 2 663 | 7.2 | 1 229 | 5.7 |
|  | Heparin (-derivates) (ATC-code B01AA) | 2 285 | 6.2 | 944 | 2.6 | 1 194 | 5.5 |
| Treatment with anti-acid drugs | | 14 596 | 39.6 | 13 122 | 35.6 | 8 218 | 38.1 |
|  | Proton pump inhibitors (PPI) (ATC code A02BC) | 14 171 | 38.5 | 12 453 | 33.8 | 7 989 | 37.0 |
|  | Histamine H2-blockers (ATC code A02BA) | 463 | 1.3 | 477 | 1.3 | 259 | 1.2 |
| Treatment with anti-depressants (ATC code N06A) | | 3 952 | 10.7 | 4 418 | 12.0 | 2 239 | 10.4 |
| Treatment with anti-diabetic drugs (ATC codes A10A, A10B) | | 5 891 | 16.0 | 6 692 | 18.2 | 3 462 | 16.0 |
| Treatment with drugs against obstructive airway disease | | 11 021 | 29.9 | 8 877 | 24.1 | 6 375 | 29.5 |
|  | Long-acting beta2 agonists (LABA) (ATC code R03AC) | 2 640 | 7.2 | 1 222 | 3.3 | 1 437 | 6.7 |
|  | Long-acting muscarinic antagonists (LAMA) (ATC code R03BB) | 4 690 | 12.7 | 2 669 | 7.2 | 2 438 | 11.3 |
|  | Inhaled corticosteroids (ICS) (ATC code R03BA) | 2 794 | 7.6 | 1 410 | 3.8 | 1 726 | 8.0 |
|  | Combination product LABA/ICS (ATC code R03AK) | 5 135 | 13.9 | 3 562 | 9.7 | 2 950 | 13.7 |
|  | Combination product LABA/LAMA (ATC code R03AL) | 40 | 0.1 | 8 | 0.0 | 25 | 0.1 |
| Treatment with PAH drugs (ATC codes B01AC09, G04BE03, CO2KX) | | 265 | 0.7 | 178 | 0.5 | 138 | 0.6 |

SA = Sensitivity analysis
