# Supplementary material for: Associations between comorbidities, their treatment and survival in patients with interstitial lung diseases – a claims data analysis
Source: Respir Res. 2018 Apr 25;19:73. doi: 10.1186/s12931-018-0769-0 (PMC5918773; doi:10.1186/s12931-018-0769-0)
Supplement: Supplementary file 8 — Table S6. Proportion of treated comorbid conditions according to sensitivity analyses. (DOC 60 kb) [file 12931_2018_769_MOESM8_ESM.doc]

Table S6: Proportion of treated comorbid conditions according to sensitivity analyses

| **Share of diagnosed individuals with comorbidity-relevant prescription at baseline** | | | | | | |
| --- | --- | --- | --- | --- | --- | --- |
|  | **Main** | | ***SA 1*** | | ***SA2*** | |
|  | **N  Diagnosed** | **Treated** | **N  Diagnosed** | **Treated** | **N  Diagnosed** | **Treated** |
| Congestive heart failure | 11 392 | 88.3 | 11 573 | 88.4 | 6 198 | 87.5 |
| Cardiac arrhythmia | 9 954 | 55.8 | 9 199 | 52.1 | 5 569 | 52.5 |
| Valvular disease | 5 433 | 74.8 | 5 626 | 75.6 | 3 053 | 71.5 |
| IHD | 12 010 | 80.1 | 12 808 | 81.4 | 6 618 | 78.4 |
| Hypertension without complications | 18 690 | 92.2 | 18 931 | 92.3 | 11 132 | 92.1 |
| Hypertension with complications | 6 653 | 90.2 | 6 775 | 90.4 | 3 315 | 90.2 |
| Pulmonary circulation disorders | 2 997 | 62.6 | 2 298 | 51.3 | 1 704 | 58.5 |
| COPD | 18 078 | 46.6 | 17 002 | 43.2 | 11 835 | 45.0 |
| Peripheral vascular disorders | 7 004 | 50.9 | 7 004 | 50.7 | 3 772 | 48.8 |
| Diabetes without complications | 6 189 | 38.0 | 6 487 | 40.9 | 3 771 | 39.2 |
| Diabetes with complications | 5 294 | 65.0 | 5 862 | 68.4 | 2 925 | 66.8 |
| Depression | 7 589 | 34.7 | 8 185 | 39.4 | 4 260 | 35.7 |
| GERD | 5 587 | 62.5 | 5 968 | 64.9 | 3 280 | 63.7 |

SA1 = Sensitivity Analysis 1, SA2 = Sensitivity Analysis 2
