# Supplementary material for: Associations between comorbidities, their treatment and survival in patients with interstitial lung diseases – a claims data analysis
Source: Respir Res. 2018 Apr 25;19:73. doi: 10.1186/s12931-018-0769-0 (PMC5918773; doi:10.1186/s12931-018-0769-0)
Supplement: Supplementary file 9 — Table S7. Associations of clinical characteristics and survival within the sensitivity analyses. (DOC 146 kb) [file 12931_2018_769_MOESM9_ESM.doc]

Table S7: Associations of clinical characteristics and survival within the sensitivity analyses

| **Variable** | | **Comorbidity-only  Cox model** | | **Drug-extended  Cox model** | |
| --- | --- | --- | --- | --- | --- |
|  |  | **SA1** | **SA2** | **SA1** | **SA2** |
| **Comorbidity profile** | Congestive heart failure treated | 1.53*** | 1.62*** | 1.48*** | 1.57*** |
|  | Congestive heart failure untreated | 1.42*** | 1.57*** | 1.34*** | 1.52*** |
|  | Cardiac arrhythmia treated | 0.95^n.s^ | not sel. | 0.95^n.s^ | not sel. |
|  | Cardiac arrhythmia untreated | 1.15*** | 1.08* | 1.15*** | 1.00^n.s^ |
|  | Valvular disease treated | 0.94** | 0.89*** | 0.94* | 0.90** |
|  | Valvular disease untreated | 1.14** | 1.26*** | 1.06 ^n.s^ | 1.12* |
|  | Pulmonary hypertension treated | 1.36*** | 1.24*** | 1.20*** | 1.16** |
|  | Pulmonary hypertension untreated | 1.46*** | 1.29*** | 1.57*** | 1.31*** |
|  | Peripheral vascular disorders treated | 1.07* | not sel. | 1.14*** | 1.07^n.s^ |
|  | Peripheral vascular disorders untreated | 1.17*** | 1.0^ns^ | 1.13*** | 1.11** |
|  | Hypertension without complications treated | 0.94* | 0.83^ns^ | 1.06^ns^ | not sel. |
|  | Hypertension without complications untreated | 1.22*** | 1.01^ns^ | 1.01^ns^ | 0.94^ns^ |
|  | Hypertension with complications treated | 0.80*** | 0.97^ns^ | 0.91^**^ | 0.97 ^s^ |
|  | Hypertension with complications untreated | 1.07 ^n.s^ | 1.26*** | 0.91*** | 0.83* |
|  | COPD treated | 0.97^n.s^ | 0.86*** | 0.91^ns^ | 0.78*** |
|  | COPD untreated | **1.09***** | 1.00^ns^ | 1.11^ns^ | **0.91***** |
|  | Diabetes without complications treated | 1.07^ns^ | 0.79** | not sel. | not sel. |
|  | Diabetes without complications untreated | 1.14*** | 0.92*** | 1.31*** | 1.20^ns^ |
|  | Diabetes with complications treated | **1.11***** | 1.06^ns^ | **0.79**** | not sel. |
|  | Diabetes with complications untreated | 1.19*** | 1.17*** | 1.15*** | 1.22^ns^ |
|  | Hypothyroidism | 0.88*** | 0.94^ns^ | 0.89^ns^ | 0.94^ns^ |
|  | Renal failure | 1.25*** | 1.32*** | 1.23*** | 1.32*** |
|  | Liver disease | 1.05* | not sel. | 1.05** | not sel. |
|  | Metastatic carcinoma | 2.30*** | 2.69** | 2.77*** | 2.63** |
|  | Solid tumour without metastasis | 1.15*** | 1.09*** | 1.09*** | 1.10*** |
|  | Rheumatoid arthritis/Connective tissue disorder | 0.91** | 0.82*** | 0.89** | 0.81*** |
|  | Coagulopathy | 1.37*** | 1.59*** | 1.34*** | 1.59*** |
|  | Obesity | 0.91*** | 0.87*** | 0.92*** | 0.89*** |
|  | Weight loss | 1.51*** | 1.58*** | 1.50* | 1.52*** |
|  | Fluid and electrolyte disorders | 1.65*** | 2.41*** | 1.62^ns^ | 2.37*** |
|  | Deficiency anaemia | 1.09* | 1.11* | 1.08^ns^ | 1.10* |
|  | Depression treated | 1.00^ns^ | 1.00^n.s^ | not sel. | not sel. |
|  | Depression untreated | not sel. | not sel. | 1.07^ns^ | not sel. |
|  | IHD treated | not sel. | not sel. | 0.95^n.s^ | 1.03^ns^ |
|  | IHD untreated | not sel. | 1.09** | 1.25*** | 1.41*** |
|  | GERD treated | 0.92** | 0.84*** | 0.89*** | 0.87*** |
|  | GERD untreated | 0.92* | 0.88* | 0.97^ns^ | 0.86** |
|  | OSAS | 0.78*** | 0.70*** | 0.78*** | 0.71*** |
|  | Lung cancer | 1.80*** | 1.93*** | not sel. | 1.92*** |
| **Medication intake** | Digitalis glycosides |  |  | 1.06^ns^ | 1.01^ns^ |
|  | Anti-arrhythmic drugs |  |  | 0.85^*^ | 0.80* |
|  | Diuretic drugs |  |  | 1.25*** | 1.16*** |
|  | Statins |  |  | 0.85*** | 0.84*** |
|  | Beta-blockers |  |  | 0.96*** | 0.89** |
|  | ACE inhibitors |  |  | 0.84^ns^ | 0.84*** |
|  | Angiotensin-I-antagonists |  |  | 0.74*** | 0.71*** |
|  | Antiplatelet drugs |  |  | 1.10*** | 1.09^**^ |
|  | Vitamin-K antagonists |  |  | 1.07^ns^ | 1.06^ns^ |
|  | Heparin (-derivates) |  |  | not sel. | 0.87** |
|  | Proton pump inhibitors |  |  | 1.05* | 0.97^ns^ |
|  | H2-antagonists |  |  | 1.24* | 1.17^ns^ |
|  | Treatment with anti-depressants |  |  | 1.15** | 1.09* |
|  | Treatment with anti-diabetic drugs |  |  | 1.39** | 1.23^ns^ |
|  | Long-acting beta2 agonists (LABA) |  |  | not sel. | 1.03^ns^ |
|  | Long-acting muscarinic antagonists (LAMA) |  |  | 1.18*** | 1.13** |
|  | Inhaled corticosteroids (ICS) |  |  | 0.93* | 0.80*** |
|  | Combination product LABA/ICS |  |  | 0.97^n.s^ | 0.94^ns^ |
|  | Combination product LABA/LAMA |  |  | not sel. | not sel. |
|  | Anti-hypertensive drugs |  |  | 1.84*** | 1.46*** |

All figures adjusted for age, gender and ILD subtype

HR = hazard ratio; not sel: not selected by LASSO

Variables with significantly positive impact shaded grey; those with impact in opposite direction in bold letters

Significance codes: p < 0.001 '***' | p < 0.01 '**' | p< 0.05 '*' | not significant ^‘ns’^.
