# Supplementary material for: Associations between comorbidities, their treatment and survival in patients with interstitial lung diseases – a claims data analysis
Source: Respir Res. 2018 Apr 25;19:73. doi: 10.1186/s12931-018-0769-0 (PMC5918773; doi:10.1186/s12931-018-0769-0)
Supplement: Supplementary file 10 — Figure S2. Comorbidity prevalence in main analysis compared with sensitivity analyses. (DOC 104 kb) [file 12931_2018_769_MOESM10_ESM.doc]

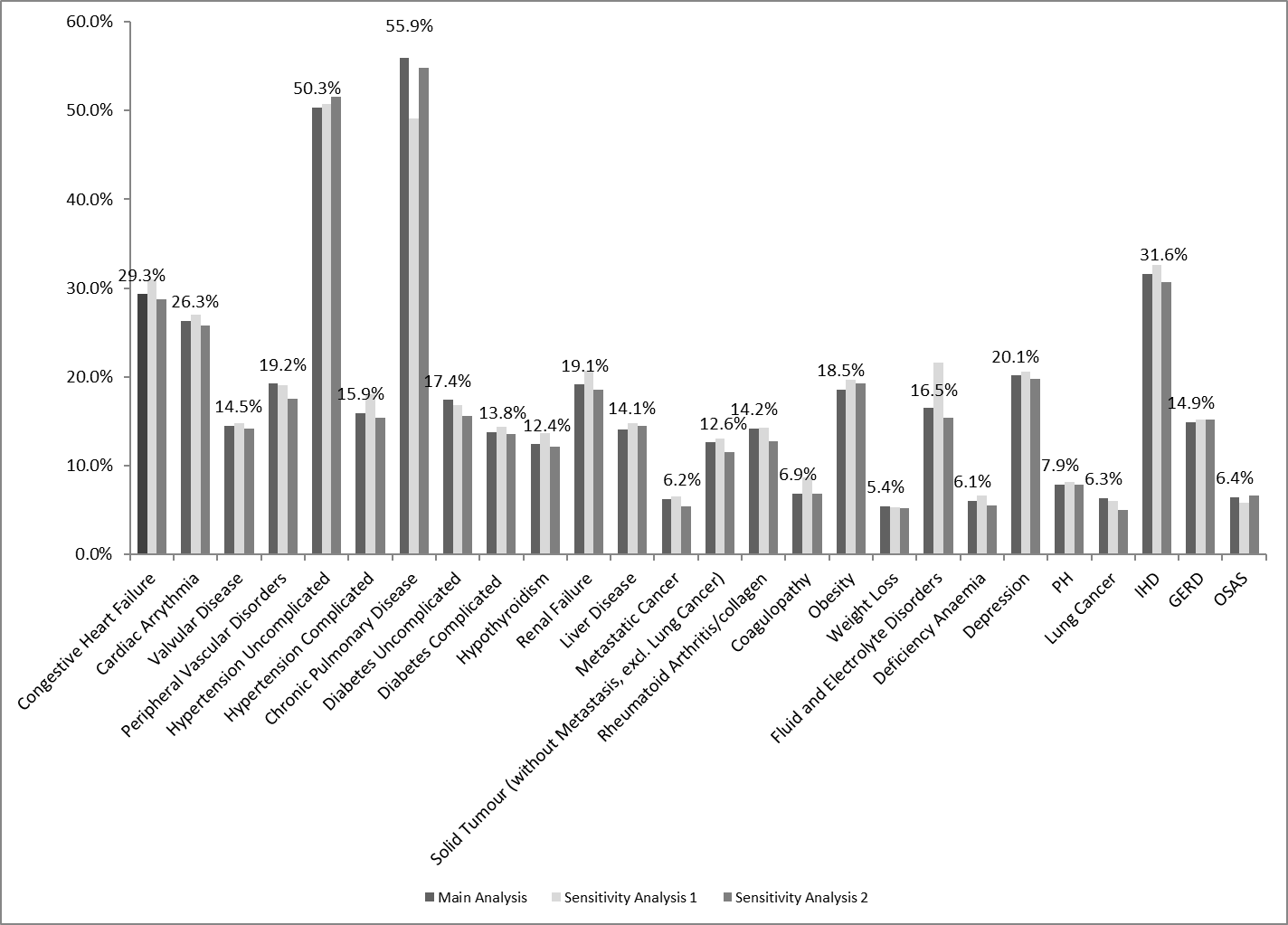

percentages refer to main analysis

Figure S2: Comorbidity prevalence in main analysis compared with sensitivity analyses
